# A Pair of Pharyngeal Gustatory Receptor Neurons Regulates Caffeine-Dependent Ingestion in *Drosophila* Larvae

**DOI:** 10.3389/fncel.2016.00181

**Published:** 2016-07-19

**Authors:** Jaekyun Choi, Lena van Giesen, Min Sung Choi, KyeongJin Kang, Simon G. Sprecher, Jae Young Kwon

**Affiliations:** ^1^Department of Biological Sciences, Sungkyunkwan University, SuwonSouth Korea; ^2^Department of Biology, Institute of Zoology, University of Fribourg, FribourgSwitzerland; ^3^Department of Anatomy and Cell Biology, Samsung Biomedical Research Institute, School of Medicine, Sungkyunkwan University, SuwonSouth Korea

**Keywords:** ingestion, bitter, caffeine, gustatory receptor neuron, *Drosophila*, larva

## Abstract

The sense of taste is an essential chemosensory modality that enables animals to identify appropriate food sources and control feeding behavior. In particular, the recognition of bitter taste prevents animals from feeding on harmful substances. Feeding is a complex behavior comprised of multiple steps, and food quality is continuously assessed. We here examined the role of pharyngeal gustatory organs in ingestion behavior. As a first step, we constructed a gustatory receptor-to-neuron map of the larval pharyngeal sense organs, and examined corresponding gustatory receptor neuron (GRN) projections in the larval brain. Out of 22 candidate bitter compounds, we found 14 bitter compounds that elicit inhibition of ingestion in a dose-dependent manner. We provide evidence that certain pharyngeal GRNs are necessary and sufficient for the ingestion response of larvae to caffeine. Additionally, we show that a specific pair of pharyngeal GRNs, DP1, responds to caffeine by calcium imaging. In this study we show that a specific pair of GRNs in the pharyngeal sense organs coordinates caffeine sensing with regulation of behavioral responses such as ingestion. Our results indicate that in *Drosophila* larvae, the pharyngeal GRNs have a major role in sensing food palatability to regulate ingestion behavior. The pharyngeal sense organs are prime candidates to influence ingestion due to their position in the pharynx, and they may act as first level sensors of ingested food.

## Introduction

Food intake, or feeding, is composed of a series of behavioral modules or subprograms (Pool and Scott, 2014) that encompass the search for food, ingestion of food, digestion, and nutrient absorption. Once the food source is confirmed as palatable, foraging stops and meal consumption/ingestion is initiated. During this process, chemical cues of the food source are continuously assessed to determine if the food source indeed qualifies for ingestion and digestion. Understanding the molecular and cellular basis of the sequential neural circuits involved in each step of feeding is still at an early stage. Foraging and ingesting food are extremely robust behaviors in *Drosophila* larvae, rendering it an ideal model to study the mechanisms of the initial feeding processes.

The larval taste system is relatively simple compared to the adult counterpart, raising the question of how larvae are able to perceive and distinguish a great multitude of distinct tastants. The major gustatory organs of *Drosophila* larvae are located in bilaterally symmetrical pairs on the head, and are composed of three external chemosensory organs: the terminal, ventral, and dorsal organs (TO, VO, and DO, respectively), and three chemosensory organs in the pharynx: the dorsal, ventral, and posterior pharyngeal sense organs (DPS, VPS, and PPS, respectively). The TO, VO, and DO are comprised of 32, 7, and 9 putative gustatory neurons, respectively, and the DPS, VPS, and PPS are comprised of ~17, 16, and 6 neurons that mostly appear to have gustatory functions (Singh and Singh, 1984; Stocker, 1994; Python and Stocker, 2002; Gendre et al., 2004; Gerber and Stocker, 2007). Gustatory neurons from these chemosensory organs project through multiple nerve tracts to the subesophageal ganglion of the larval brain (Stocker, 1994; Python and Stocker, 2002; Gendre et al., 2004; Colomb et al., 2007; Vosshall and Stocker, 2007; Kwon et al., 2011).

Members of the *Gustatory receptor* (Gr; Colomb et al., 2007; Thorne and Amrein, 2008; Kwon et al., 2011; Mishra et al., 2013; van Giesen et al., 2016), *Ionotropic receptor* (Ir; Stewart et al., 2015), and *pickpocket* (*ppk*; Liu et al., 2003) families are involved in chemosensory perception, and expressed in the larval gustatory neurons. For the 68 Grs, 39 *Gr-GAL4* drivers were shown to express in the major taste organs of the larval head. A receptor-to-neuron map was constructed for 28 Grs expressed in 10 gustatory receptor neurons (GRNs) in the terminal organ and dorsal organ. These GRNs were designated the DO group (A1 and A2), TO-dorsolateral group (B1 and B2), and TO-distal group (C1-6) based on cell body position (Kwon et al., 2011). Although the pharyngeal sense organs house close to half of the putative gustatory neurons in the larval head, surprisingly little is known about their function.

Here, through comprehensive analysis, we construct a detailed receptor-to-neuron map of Gr gene expression in the pharyngeal organs. By combining molecular genetic tools, behavioral assays, and genetically coded calcium sensors to assess neuronal activity, we show that a specific pair of GRNs in the pharyngeal sense organs, DP1, has a major role in caffeine-driven ingestion in *Drosophila* larvae.

## Materials and Methods

### *Drosophila* Stocks and Transgenes

Flies were cultured on standard cornmeal agar medium at room temperature (23 ± 2°C). All *Gr-GAL4* transgenic lines used in this study were previously described (Kwon et al., 2011). *wCS* was used a control for behavioral assays. To construct the *Gr33a-QF* transgene, 1,217 bp of the 5′ upstream region of the *Gr33a* gene was amplified using the 5′-CGGATCCCCTTGGTCAAAAATA-3′ and 5′-CGAATTCATTGCTCGGAATTTACTCGCTAC-3′ primers, and cloned into the pattB-QF-hsp70 vector. The following fly lines were used: *QUAS-mtdTomato* (Potter et al., 2010), *orco^1^*(Larsson et al., 2004), *Gr33a^1^*(Moon et al., 2009), *UAS-Kir2.1* (Baines et al., 2001), *UAS-TNT* (Sweeney et al., 1995), *UAS-Gr33a* (Moon et al., 2009), *UAS-GCaMP5* (Akerboom et al., 2013).

### *Gr-GAL4* Expression Mapping in the Pharyngeal Sense Organs

*Gr-GAL4* drivers used in a previous study (Kwon et al., 2011) were used to map expression in the pharyngeal sense organs in a manner similar to the mapping of *Gr-GAL4* driver expression in the terminal organ at the cellular level (Kwon et al., 2011). For each *Gr-GAL4* transgene, we used the line with the most penetrant expression that had been selected as a representative line (Kwon et al., 2011). Twenty-third instar larvae containing two copies each of the *Gr-GAL4* and *UAS-mCD8-GFP* transgenes were observed for expression in the pharyngeal sense organs. To determine whether two *Gr-GAL4* drivers were expressed in the same cells, larval progeny from crosses between two *Gr-GAL4; UAS-mCD8-GFP* strains were observed for GFP expression, to count whether the number of GFP-expressing cells was unchanged or increased compared to the parent strains. For homozygote lethal *Gr-GAL4* strains (*Gr22b-*, *Gr28a-*, *Gr57a-*, *Gr58b-*, *Gr59d-*, and *Gr66a-GAL4*), a balancer containing GFP was used to distinguish progeny containing both *Gr-GAL4* drivers. To quantitate expression of each *Gr-GAL4* line and the combinations of *Gr-GAL4* drivers, we counted the number of GFP-labeled cells on both the left and right sides of all larvae and calculated the average number of labeled cell pairs. For example, if we examined 20 larvae of a particular *Gr-GAL4* line and observed that fifteen animals expressed GFP in one dorsal pharyngeal neuron on each side (15 × 2 = 30), four animals expressed GFP in only one neuron on one side (4 × 1 = 4), and one animal did not show GFP expression (1 × 0 = 0), the maximum number of labeled dorsal pharyngeal neurons is 1 (**Table 1**), and the average expression level is (30 + 4 + 0)/40 = 0.85 (**Supplementary Table S1**).

**Table 1:** Summary of *Gr-GAL4* expression patterns in the pharyngeal sense organs.

	Pharyngeal organs			X *Gr2a -GAL4*
	DPS	VPS	PPS	DPS
*Gr2a*	1(1)	-	-	1
*Gr9a*	1	-	-	2
*Gr22b*	1	-	2-4	2
*Gr22d*	1	-	-	2
*Gr22e*	1	-	-	2
*Gr23a*	1(1)	-	-	1
*Gr28a*	-	2-3(2-3)	2-4(2)	-
*Gr28b.a*	1	-	-	2
*Gr32a*	2	-	2-3	3
*Gr33a*	2	2	2-4	3
*Gr39a.a*	1	-	3-4	2
*Gr39a.b*	1	-	-	2
*Gr39a.d*	-	-	1-2	-
*Gr39b*	1	-	1-3	2
*Gr43a*	1(1)	-	-	2
*Gr57a*	1(1)	-	-	1
*Gr58b*	1	-	-	2
*Gr59d*	1	-	-	2
*Gr66a*	2	2	1-4	3
*Gr68a*	-	3(1)	-	-
*Gr77a*	1	-	-	2
*Gr93a*	2(1)	-	-	3
*Gr93b*	1	-	-	2
*Gr93c*	-	-	1-2	-
*Gr93d*	1	-	1-2(1)	1

*Numbers indicate the maximum number (DPS) or range (VPS and PPS) of neurons labeled on one side of the animal by the indicated *Gr-GAL4* driver or the driver crossed to *Gr2a-GAL4*. Numbers in parentheses indicate the number of neurons that do not overlap with *Gr33a-QF*, *QUAS-mtdTomato* expression.*

The QF-QUAS system (Potter et al., 2010) was also used. In detail, we first mapped the *Gr-GAL4* drivers expressed in each pharyngeal sense organ to individual neurons using the Q system (**Table 1**). We observed at least 20 larvae for each genotype. First, we generated the broadly expressing *Gr33a-QF*, and examined larvae containing one copy each of *Gr33a-QF*, *QUAS-mtdTomato*, *GrX-GAL4*, and *UAS-mCD8-GFP* to determine whether *GrX-GAL4* is expressed in the same neurons as *Gr33a-GAL4*-expressing neurons. Through this analysis, we found that in the DPS, *Gr2a*-, *Gr23a*-, *Gr43a*-, and *Gr57a*-*GAL4* show expression independent of *Gr33a-GAL4*, and one of the two pairs of *Gr93a-GAL4*-expressing DPS neurons expresses independently of *Gr33a-GAL4* while the other pair co-localizes with *Gr33a-GAL4*. *Gr93d-GAL4* is also expressed independently of *Gr33a-GAL4* in the DPS, but single copies of the *GAL4* and *UAS-GFP* transgenes during double labeling with the Q system resulted in low GFP intensity that was too faint to visualize. Similarly, we were not able to visualize the double labeling results for *Gr9a-* and *Gr93b-GAL4* in the DPS and *Gr39a.d-GAL4* in the PPS, and thus we relied on the results of different combinations of crosses with other *Gr-GAL4* drivers to construct a GRN map. In the VPS, all *Gr28a-GAL4*- and one pair of *Gr68a-GAL4*-expressing cells are expressed independently of *Gr33a-GAL4*. In the PPS, some *Gr28a-GAL4*- and *Gr93d-GAL4*-expressing cells do not co-express with *Gr33a-GAL4*. Next, we took the *Gr2a-GAL4* driver, which was observed to express independently of *Gr33a-GAL4* in the DPS, and examined co-expression with other *Gr-GAL4* drivers in the DPS (**Table 1**). Through these experiments, the expression of *Gr-GAL4* drivers in the two pairs of *Gr33a-GAL4*-expressing cells in the DPS were re-confirmed, since an additive number of neuron pairs was observed in combination with the *Gr2a-GAL4* driver (**Supplementary Table S1**). Three *Gr-GAL4* drivers (*Gr23a*-, *Gr57a*-, and *Gr93d-GAL4*) that were expressed in cells independent of *Gr33a-GAL4* were observed to co-express with *Gr2a-GAL4*, since the number of cells expressing in the DPS was unchanged upon combination with the *Gr2a-GAL4* driver. In contrast, *Gr43a-GAL4* was observed to express independently of *Gr2a-GAL4* as well as *Gr33a-GAL4*. Through extensive examination of different combinations of *Gr-GAL4* drivers in this manner, a comprehensive Gr-GRN map of the pharyngeal sense organs was constructed.

The expression maps of the VPS and PPS were constructed mainly based on the *Gr33a-QF* results. The number of *Gr-GAL4*-expressing cells in the VPS and PPS varies greatly from individual to individual, rendering it difficult to use combinations of *Gr-GAL4* lines to directly observe neurons co-expressing certain drivers, for example *Gr28a-GAL4* and *Gr68a-GAL4* in VP3.

### Behavioral Assay

Feeding stage third instar larvae were used for all behavioral assays. Experiments were conducted with larvae from vials 5 days after egg-laying. Larvae were washed three times with distilled water before experiments.

For the ingestion assay, previous protocols were basically followed with some modifications (Schipanski et al., 2008; Rohwedder et al., 2012). Briefly, 60 mm petri dishes (SPL 10060) were filled with 1% agarose solution + 1% indigo carmine (Sigma, 57000; control plates), or 1% agarose solution + 1% indigo carmine + bitter substance (experimental plates). Thirty-third instar larvae were placed in the center of the plate. Ninety minutes was selected as the assay period because the amount of dye ingested by *wCS* larvae was observed to saturate at 90 min. After 90 min of feeding, the larvae were collected and washed with distilled water. After washing, larvae were homogenized in 1 M 60 μl L-ascorbic acid (Sigma, A7506) using a pistil to prevent discoloration due to debris from the homogenized larvae. Following centrifugation at 13,200 rpm for 10 min, the blue supernatant was transferred to a 1 μm-pore FAPD column (FAVORGEN, BCP01-1-100) and centrifuged at 13,200 rpm for 3 min for filtration. The filtered supernatant was transferred to a 96-well plate (SPL 30096) and absorbance was measured at 630 nm using a spectrophotometer (BioTek EL800). The relative ingestion index (I.I.) was derived by calculating the difference in absorbance between the control and experimental groups: I.I. = [(experimental OD–empty OD) – (dye only control OD–empty OD)]/(dye only control OD–empty OD). The dye only control OD was measured at every experiment, for every genotype, on the same day. For example, if the ingestion of *wCS* larvae in response to 10 mM caffeine was tested, larvae from the same vial would be washed and 30 larvae each would be placed on a dye only plate and 10 mM caffeine-containing plate at the same time to feed. After processing the fed larvae, each OD value would be measured to obtain one *n*-value for the I.I. The empty OD indicates a blank measurement of the OD of larvae fed on an agarose plate without indigo carmine, and showed a consistent value of 0.03 in our conditions (OD = 0.031 ± 0.0016, *n* = 18). In our ingestion assays, we measured a minimum OD value of 0.03 (when larvae did not ingest any food), which corroborated the blank measurement, and a maximum value of 0.45. To test whether the OD values that we measure can represent the amount of ingested dye in a linearly proportional manner, we measured the OD of solutions with different known amounts of indigo carmine dissolved in ascorbic acid. When indigo carmine was added in 0.01 mg increments to ascorbic acid in the range of 0.01 mg/ml (OD 0.049) to 0.1 mg/ml (OD 0.392), the OD increased in a completely linear pattern (**Supplementary Figure S1**). When indigo carmine was added to ascorbic acid at higher concentrations, 0.5 mg/ml (OD 1.896), 1 mg/ml (OD 3.1905), 5 mg/ml (OD 3.662), 10 mg/ml (OD 3.435), it appeared that the OD saturated at around OD 3 (**Supplementary Figure S1**). Thus, in the range of OD values that we measured during our assays (0.03 to 0.45), the OD value appears to be linearly proportional to the amount of dye ingested by larvae. An I.I. value of 0 indicates that larvae on the test plate ate as much as larvae on the dye only control plate, while I.I. = -1 indicates that larvae on the test plate did not eat at all, and I.I. > 0 indicates that larvae on the test plate ate more than larvae on the dye only control plate.

### Fluorescence Imaging and Immunostaining

For imaging *UAS-mCD8-GFP* and *QUAS-mtdTomato* expression in the pharyngeal sense organs, larval heads were dissected and incubated in mounting solution (50% glycerol in 1X PBS-T) for 20–30 min before direct observation of fluorescence.

For the immunostaining of larval brain projections, larval brains were dissected and immunostained as previously described (Kwon et al., 2011). Anti-GFP (rabbit polyclonal; Invitrogen; 1:1,000 dilution) was used to amplify the GFP signal of *UAS-mCD8-GFP* in *GAL4*-expressing cells. Anti-nc82 (mouse monoclonal; a gift of Dr. Alois Hofbauer, University of Regensburg; 1:100 dilution) was used to visualize a presynaptic active zone protein that marks brain morphology. The secondary antibodies used were goat anti-rabbit IgG conjugated to Alexa 488 (Invitrogen; 1:1,000) and goat anti-mouse conjugated to Alexa 568 (Invitrogen; 1:1,000). All images were taken using a confocal microscope (Zeiss LSM 510 or LSM 700).

### GCaMP Imaging

To record calcium responses, early stage third instar larvae were dissected in modified AHL-Saline (NaCl, 108 mM; KCl, 5 mM; MgCl_2_, 8.2 mM; NaHCO_3_, 4 mM; NaH_2_PO_4_, 1 mM; HEPES, 5 mM; pH 7.5, in Millipore water). After the cuticle surrounding the tip of the head was removed to enable recording in DP1 and DP2, the head was introduced into the chamber, to assure exposure of chemosensory organs to the liquid passing through the channel. A drop of 2% agarose diluted in AHL saline was used to close the channel. Measurements were carried out as followed: a 10 s period of washing substance (Millipore water) followed by a 20 s period of stimulation and another 10 s of washing. Changes in fluorescence were calculated as follows: Δ*F*/*F* (%) = (*F*_peak_-*F*_0_)*100/*F*_0_ *F*_0_ was calculated from five frames during the unstimulated phase of the first 100-frame time period. *F*_peak_ was taken as the point of highest intensity measured during the time of stimulation. For the analysis of calcium imaging measurements, LASAF Software (Leica) was used and changes in fluorescence were calculated in Microsoft Excel 14.4.5. Statistical analysis was performed using RStudio 0.98.1087. Error bars indicate SEM and comparison between two samples was performed by using the Wilcoxon signed-rank test and Student’s *t*-test to verify significant differences from 0.

### Statistics

All statistical analysis was performed using the statistics program IBM SPSS Statistics 20. All behavior data are presented as a box plot, with the middle line representing the median, the ‘+’ the mean, and the box boundaries and whiskers representing 25%/75% and 10%/90%, respectively. *Kruskal–Wallis* tests were used for multiple comparisons of various genotypes. The *Mann–Whitney U* test was used for pair-wise comparison. Asterisks shown in figures signify statistical significance (*p < 0.05, **p < 0.01). Additional details are described in the figure legends.

## Results

### Construction of a Comprehensive Pharyngeal Gustatory Receptor-to-Neuron Map

**FIGURE 1:**
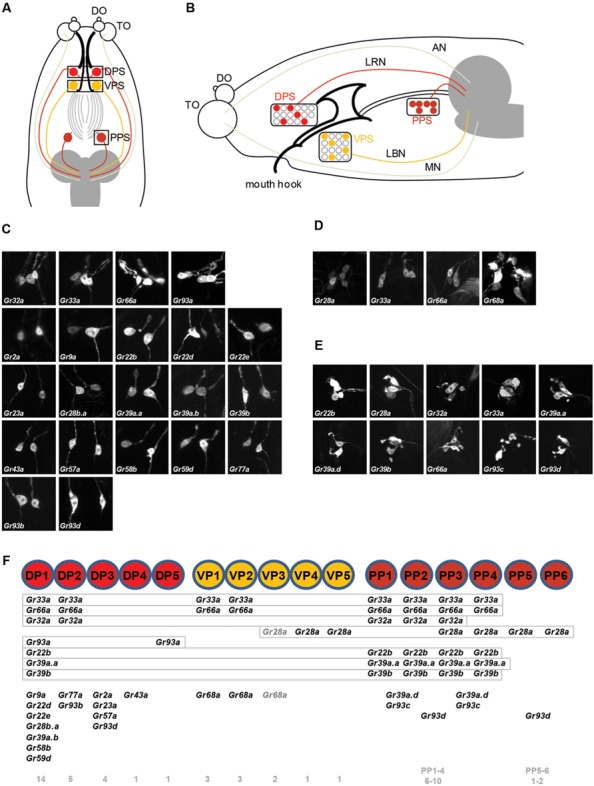
**A gustatory receptor-to-neuron map in the pharyngeal sense organs of *Drosophila* larva. (A,B)** The taste system of the larval head. **(A)** shows a dorsal view, with anterior to the top and **(B)** shows a lateral view, with anterior to the left. The ventral organs and ganglia of the taste organs were omitted from the schematics for simplicity. The boxes in **(A)** represent the positions at which micrographs were taken for the DPS **(C)**, VPS **(D)**, and PPS **(E)**. The DPS was drawn anterior to the VPS for convenience of representation, and does not reflect the actual relative positions of the DPS and VPS. The colored pharyngeal sensory neurons in **(B)** represent the neurons for which gustatory receptor expression was mapped **(F)** and the positions of the cell bodies in the diagram are arbitrary. Among the neurons that innervate the TO, the TO-dorsolateral group projects through the antennal nerve and the TO-distal group projects through the maxillary nerve to the larval brain. DO, dorsal organ; TO, terminal organ; DPS, dorsal pharyngeal sensilla; VPS, ventral pharyngeal sensilla; PPS, posterior pharyngeal sensilla; AN, antennal nerve; LRN, labral nerve; LBN, labial nerve; MN, maxillary nerve. **(C–E)** Expression of *Gr-GAL4* drivers in the pharyngeal sense organs. **(C)** The *Gr32a-*, *Gr33a-*, *Gr66a-*, and *Gr93a-GAL4* drivers are expressed in two pairs of neurons in the DPS, and the remaining 17 *Gr-GAL4* drivers are expressed in a single pair of neurons in the DPS. **(D)** *Gr28a-* and *Gr68a-GAL4* are expressed in three pairs of neurons in the VPS, and *Gr33a-* and *Gr66a-GAL4* are expressed in two pairs of neurons in the VPS. **(E)** Ten *Gr-GAL4* drivers showed GFP reporter expression in 1–4 pairs of neurons in the PPS, with weak and inconsistent expression from individual to individual. **(F)** Pharyngeal gustatory receptor (Gr)-to-neuron map. Co-expressed *Gr-GAL4* drivers are listed for each neuron. The numbers on the bottom show the numbers of *Gr-GAL4* transgenes expressed in each pharyngeal GRN. The number of *Gr-GAL4*-expressing cells in the VPS and PPS varies greatly from individual to individual, rendering it difficult to use combinations of *Gr-GAL4* lines to directly observe neurons co-expressing certain drivers. Gray letters were used for *Gr28a-GAL4* and *Gr68a-GAL4* because we could not be sure that they co-express in VP3. Also, although we observed that PPS-expressing *Gr39a.d*-, *Gr93c*-, and *Gr93d-GAL4* co-expressed with *Gr33a-GAL4* expressing neurons, we could not verify co-expression between those transgenes, and so the transgenes were marked in a range underneath the PPS cells, rather than in definitive positions.

To examine the roles of specific Grs and GRNs in the regulation of feeding behavior, comprehensive Gr-to-GRN maps of the head sensory organs of larvae are important. Since a detailed map of the external sensory organs existed (Kwon et al., 2011), we further constructed a comprehensive Gr-to-GRN map of the pharyngeal sense organs using the *GAL4/UAS* system and the Q system (Potter et al., 2010). Twenty-four *Gr-GAL4* drivers were previously reported to be expressed in the pharyngeal sense organs (Kwon et al., 2011), but a detailed receptor-to-neuron map was not constructed. We first re-analyzed all individual 68 *Gr* genes using the *GAL4/UAS* system, with an emphasis on expression in the pharyngeal sense organs. The DPS, VPS, and PPS, which compose the pharyngeal sense organs, each contain ~17, 16, and 6 neurons, respectively (**Figures 1A,B**). We confirmed the previous results and extended the sensory neuron map, while updating details on a couple of points. *Gr9a-GAL4* expression was observed in the DPS in addition to the C1 neuron in the terminal organ, and *Gr93d-GAL4* expression was observed in one pair of neurons in the DPS in addition to the previously reported expression in the PPS. *Gr93a-GAL4* expression was observed in up to two pairs of neurons in the DPS. Since expression in the VPS and PPS varied largely between animals, we determined the range of expressing cells (**Table 1**). Through additional extensive combinatorial examination using the Q system (Potter et al., 2010) and combinations of *Gr-GAL4* drivers (**Supplementary Figure S2**), we constructed a comprehensive Gr-GRN map of the pharyngeal sense organs (see Materials and Methods for details of analysis and construction). To briefly summarize, 21 *Gr-GAL4* drivers are expressed in the DPS with *Gr32a*-, *Gr33a*-, *Gr66a*-, and *Gr93a-GAL4* expressed in two pairs of neurons, and the rest in one pair (**Figures 1C,F**). Four *Gr-GAL4* drivers (*Gr28a*-, *Gr33a*-, *Gr66a*-, and *Gr68a-GAL4*) are expressed in 2–3 pairs of neurons in the VPS (**Figures 1D,F**), while 10 *Gr-GAL4* drivers are expressed in 1–4 pairs of neurons in the PPS (**Figures 1E,F**).

**FIGURE 2:**
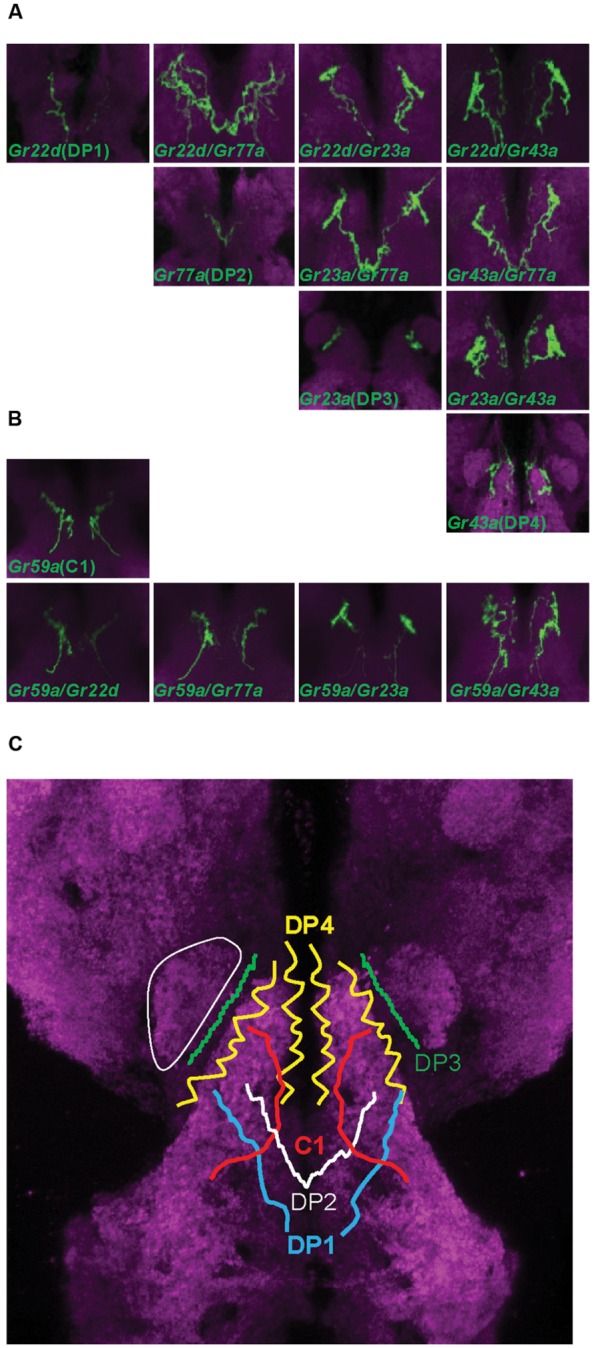
**Projection patterns of dorsal pharyngeal GRNs in the larval brain. (A)** Four *Gr-GAL4* drivers that each express in a single pair of GRNs in the DPS were individually analyzed for projection patterns in the SOG of the larval brain, and also in pairs for double-driver analysis. The brain neuropil is counterstained with the monoclonal antibody nc82 (magenta). **(B)** Projection pattern of the *Gr59a-GAL4* expressing GRN that specifically expresses in the C1 neuron of the terminal organ, and projection patterns of pairs of different dorsal pharyngeal GRN-expressing *GAL4* lines with *Gr59a-GAL4*. **(C)** Schematic drawing of a projection map of taste information relayed to the larval brain. The relative position of each neuron is depicted based on the results of **(A)** and **(B)**. One of the larval antennal lobes, which exist on both sides, is outlined with a thin solid white line.

Gustatory neurons in the head of *Drosophila* larva project to the subesophageal ganglion, the gustatory center of the larval brain. Neurons of the TO-distal group of neurons project through the maxillary nerve, and neurons in the pharyngeal sense organ project through the labral and labial nerve (**Figure 1B**; Gendre et al., 2004; Colomb et al., 2007; Kwon et al., 2011). We observed the brain projection patterns of each of the DP1-4 neurons of the DPS (**Figure 2A**), and constructed a diagram of projection patterns by comparing the relative positions of neuronal projections (**Figure 2C**). The V-shaped projection of the axon terminal of the DP2 neuron (*Gr77a-GAL4*) is positioned more anteriorly compared to DP1 (*Gr22d-GAL4*; **Figure 2C**). The axon terminal of the DP4 neuron splits into two branches and projects to the most anterior part of the SOG, while the projection of the DP3 neuron is positioned between the larval antennal lobe (LAL) and DP4 projection (**Figure 2C**). These projection patterns of the DPS GRNs are distinct from the projection pattern of the C1 neuron, observed by expression driven by the terminal organ C1 neuron-specific *Gr59a-GAL4* driver (**Figure 2B**).

### Different Putative Bitter Tastants Elicit Distinct Responses in Ingestion Behavior

**FIGURE 3:**
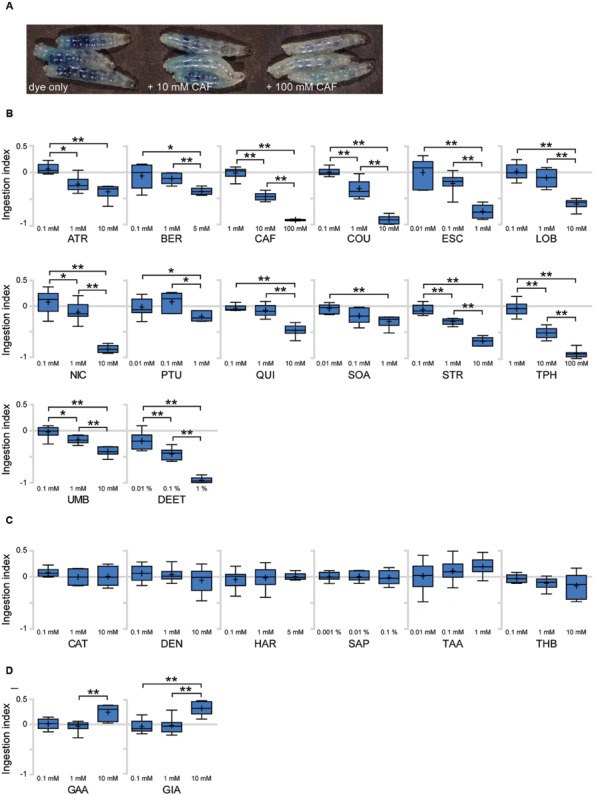
**Various responses toward putative bitter tastants in larval ingestion. (A)** Representative image showing larvae fed for 90 min on agarose plates containing indigo carmine dye only, 10 mM caffeine, and 100 mM caffeine (from left, in order). **(B–D)** Each compound was tested for ingestion at three concentrations, and three pair-wise combinations of these results (lowest concentration vs. middle concentration, middle concentration vs. highest concentration, lowest concentration vs. highest concentration) were subjected to *Mann–Whitney U* test pair-wise comparisons. Asterisks signify statistical significance (*p < 0.05, **p < 0.01). **(B)** Compounds that showed a decrease in ingestion with *p* < 0.01 at least once among the three pair-wise comparisons. **(C)** Compounds that do not show *p* < 0.05 in any of the three pair-wise comparisons. **(D)** Compounds that showed an increase in ingestion with *p* < 0.01 at least once among the three pair-wise comparisons. Each data point was derived from *n* > 6. Abbreviations stand for the following chemicals: atropine (ATR; A0132, Sigma), berberine chloride (BER; B3251, Sigma), caffeine (CAF; 27600, Sigma), coumarin (COU; C4261, Sigma), escin (ESC; E1378, Sigma), (-)-lobeline hydrochloride (LOB; 141879, Aldrich), (-)-nicotine (NIC; 36733, Fluka), *N*-phenylthiourea (PTU; P7629, Sigma), quinine hydrochloride dihydrate (QUI; 22630, Sigma), *D*-(+)-sucrose octaacetate (SOA; 84112, Fluka), strychnine nitrate (STR; S0093, TCI), theophylline anhydrous (TPH; 103024, MP Bio), umbelliferone (UMB; 93979, Sigma), *N,N*-diethyl-*m*-toluamide (DEET; PS-902, Supelco), (+)-catechin (CAT; ALX-385-017, Enzo), denatonium benzoate (DEN; D5765, Aldrich), harmaline (HAR; 51330, Aldrich), saponin (SAP; 102855, MP Bio), tannic acid (TAA; 194859, MP Bio), theobromine (THB; T4500, Sigma), gallic acid (GAA; G7384, Sigma), gibberellic acid (GIA; 63492, Aldrich).

To identify bitter tastants that affect ingestion in *Drosophila* larva, we tested 22 putative bitter compounds for effects on ingestion. Most of the tested compounds were previously used in studies on bitter sensing in adult *Drosophila* (Weiss et al., 2011) and larvae (Kim et al., 2016). To quantify larval ingestion behavior, we used agarose plates containing dye only or a specific tastant combined with dye, allowing comparison and quantification of the amounts of ingested dye. When larvae are placed on a dye-containing agarose plate for 90 min, the ingested dye is visible by eye in the digestive tract of whole larvae. Addition of increasing concentrations of bitter tastants such as caffeine results in a visible decrease of ingested agarose (**Figure 3A**). To quantify the relative amounts of ingested dye, the larvae were further processed to obtain an OD value and resulting ingestion index (I.I.) value (**Supplementary Table S2**; see Materials and Methods for details). An I.I. of 0 indicates that larvae ingested the same amount as control larvae, and an I.I. of -1 indicates that larvae did not ingest at all. An I.I. larger than 0 indicates that the tastant is a positive effector of ingestion, while an I.I. between 0 and -1 indicates a negative effector. Among the 22 putative bitter tastants tested, 14 tastants caused a decrease in ingestion in a dose-dependent manner (**Figure 3B**). Six tastants showed no difference from control plates even at the highest concentrations tested (**Figure 3C**), and two tastants caused a slight increase in ingestion at higher concentrations (**Figure 3D**).

We used the anosmic *orco* mutant (*orco^1^*; Larsson et al., 2004) to test whether the effects of tastants on ingestion are due to olfaction. When *orco^1^* mutant larvae were tested for responses to CAF, LOB, QUI, TPH, COU, ESC, and NIC, a decrease in ingestion similar to control larvae was observed, with the exception of COU (**Supplementary Figure S3**). Thus, we decided to exclude COU from further behavioral experiments, since COU perception likely includes an olfactory component.

### Pharyngeal GRNs Are Necessary and Sufficient for Caffeine-Driven Ingestion Response

To further investigate the molecular and cellular basis of ingestion control by bitter tastants, seven tastants were selected for further experiments. We selected CAF, LOB, QUI, TPH, STR, ESC, and NIC as bitter tastants that elicit an aversive response in ingestion in a dose-dependent manner. First, we examined whether ingestion reduction by bitter tastants is dependent on *Gr33a-GAL4*-expressing sensory cells. Gr33a was identified as an important co-receptor in adult bitter-sensitive neurons that act in sensing most non-volatile bitter chemicals, and the *Gr33a^1^* mutant fly is insensitive to most bitter chemicals (Moon et al., 2009). Since *Gr33a* enhancer lines are expressed in the terminal organ and pharyngeal sense organs of the larval head (Colomb et al., 2007; Kwon et al., 2011) and was shown to be expressed by RNAseq in the terminal organ ganglion (van Giesen et al., 2016), we utilized the available receptor-to-neuron maps (**Figure 1F**; Kwon et al., 2011) to examine the cellular basis of Gr33a-dependent ingestion reduction by caffeine.

**FIGURE 4:**
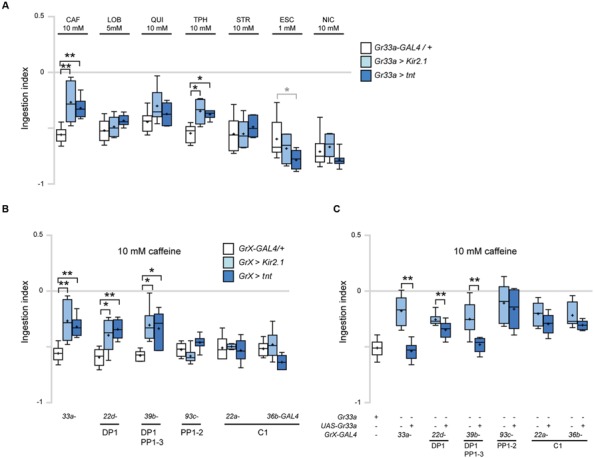
**DP1 is necessary and sufficient for caffeine-induced aversive response in ingestion. (A)** Ingestion in response to the indicated bitter tastants at various concentrations upon inhibition of the activity of *Gr33a-GAL4*-expressing GRNs. For each data, 6 < *n* < 7. *Kruskal–Wallis* test was used for multiple comparisons. Each *GrX* > *Kir2.1* and *GrX* > *TNT* was compared with the *GrX-GAL4/+* control. *p < 0.05, **p < 0.01, *p*-values of the points marked with asterisks are, 10 mM CAF, *Gr33a* > *Kir2.1*, *p* = 0.006, *Gr33a* > *TNT*, *p* = 0.007; 10 mM TPH, *Gr33a* > *Kir2.1*, *p* = 0.017, *Gr33a* > *TNT*, *p* = 0.017; 1 mM ESC, *Gr33a* > *TNT*, *p* = 0.045. *Gr33a* > *TNT* larvae in response to 1 mM ESC was marked in a gray asterisk to distinguish it from other data, since ingestion was decreased compared to other data. **(B)** Comparison of ingestion in response to 10 mM caffeine when *Gr-GAL4* drivers specifically expressed in the terminal organ and pharyngeal organs were used to block GRN activity in specific organs. For each data, 6 < *n* < 8. *Kruskal–Wallis* test was used for multiple comparisons. Each *GrX* > *Kir2.1* and *GrX* > *TNT* was compared with the *GrX-GAL4/*+ control. *p < 0.05, **p < 0.01, *p*-values of the points marked with asterisks are, *Gr33a* > *Kir2.1*, *p* = 0.006; *Gr33a* > *TNT*, *p* = 0.007; *Gr22d* > *Kir2.1*, *p* = 0.033; *Gr22d* > *TNT*, *p* = 0.005; *Gr39b* > *Kir2.1*, *p* = 0.015, *Gr39b* > *TNT*, *p* = 0.021. **(C)** Ingestion in response to 10 mM caffeine upon expression of Gr33a in the *Gr33a* mutant using the indicated *Gr-GAL4* drivers for specific expression in the GRNs listed under the underlines. “+” and “-” indicate whether the *Gr33a* or transgenes are present or absent. For each data, 6 < *n* < 21. **p < 0.01, *Mann–Whitney U* test pair-wise comparisons of *Gr33a^1^;GrX-GAL4/*+ and *Gr33a^1^;GrX* > *UAS-Gr33a*. The *p*-values of the points marked with asterisks are, *Gr33a-GAL4*, *p* = 0.002; *Gr22d-GAL4*, *p* = 0.006; *Gr39b-GAL4*, *p* = 0.005.

We first silenced neuronal activity in *Gr33a* GRNs, by expressing the inward-rectifier potassium channel UAS*-Kir2.1* or tetanus toxin *UAS-TNT* under the control of *Gr33a-GAL4* (Sweeney et al., 1995; Baines et al., 2001). Inhibiting the activity of *Gr33a-GAL4*-expressing neurons caused a decrease in CAF- and TPH-induced ingestion reduction, but did not affect LOB-, QUI-, STR-, ESC-, or NIC-induced ingestion reduction (**Figure 4A**). These results suggest that the sensing of CAF and TPH and elicitation of related behavior in larvae are mediated by a common mechanism, consistent with results in adults in which CAF and TPH both show a *Gr66a*-dependent physiological response (Moon et al., 2006).

Next, we used individual *Gr-GAL4* drivers to express either *UAS-Kir2.1* or *UAS-TNT* to block neuronal activity in specific GRNs. *GAL4* drivers may cause leaky ectopic expression that is not discernable as visible GFP reporter expression, confounding the interpretation of experimental results of behavioral assays. To additionally confirm the specificity of each *Gr-GAL4* driver, we examined the expression of *Gr-GAL4* lines in the larval head and larval central nervous system to check expression in the CNS or peripheral nervous system that projects to the CNS, and did not observe expression. Genetic silencing of neurons in the pharyngeal sense organs using *Gr22d-* and *Gr39b-GAL4* caused a decrease in caffeine-induced ingestion reduction similar to the effects of using *Gr33a-GAL4* (**Figures 4A,B**). In contrast, no changes in ingestion were observed when neurons expressing pharyngeal GRNs such as PP1-2, or terminal organ-specific *Gr-GAL4* drivers were inhibited (**Figure 4B**). We also observed that the *Gr33a^1^* mutant is defective in caffeine-induced ingestion, and these defects are rescued by a *UAS-Gr33a* transgene (**Figure 4C**). Interestingly, when *Gr22d-* and *Gr39b-GAL4*, expressed in DP1, DP1 and PP1-3, respectively, were used to specifically express Gr33a in the pharyngeal sense organs, the *Gr33a^1^* mutant phenotype was rescued (**Figure 4C**). In contrast, no rescue was observed when *GAL4* drivers expressed in pharyngeal GRNs such as PP1-2, or terminal organ-specific drivers such as *Gr22a*- and *Gr36b*-*GAL4* were used to drive Gr33a expression (**Figure 4C**). These results collectively indicate that *Gr22d-* and *Gr39b-GAL4*-expressing pharyngeal GRNs are necessary and sufficient for caffeine-driven ingestion, and that DP1 is likely the major neuron that acts in caffeine sensing.

### The DP1 Neuron Pair Responds to Caffeine

**FIGURE 5:**
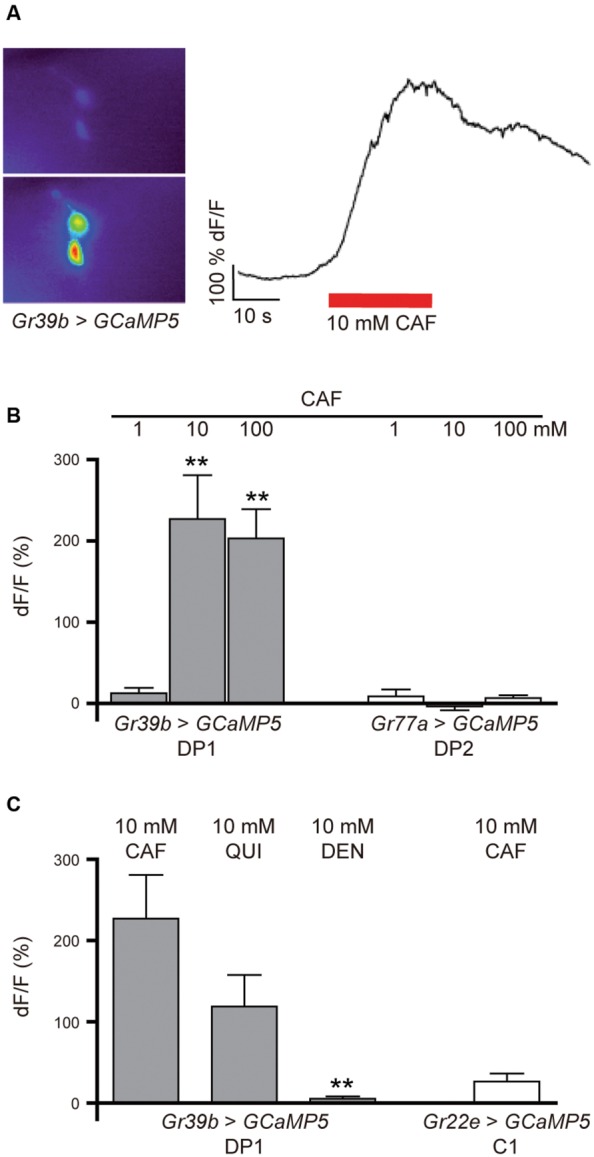
**Calcium response in DP1, DP2, and C1 after stimulation with bitter substances. (A)** Calcium currents can be measured in the pharyngeal neurons before and during the application of tastants using the genetically encoded calcium sensor GCaMP5. **(B)** Pharyngeal DP1 neurons labeled by *Gr39b-Gal4* showed concentration-dependent neuronal activity to 10 and 100 mM caffeine (Student’s *t*-test, CAF 10 mM, *p* = 0.003; 100 mM, *p* = 0.000; *n* = 5–6). The DP2 neuron pair labeled by *Gr77a-Gal4* showed no response to the applied concentrations of caffeine (*n* = 4). **(C)** The DP1 neurons also responded to 10 mM quinine at a lower average of fluorescent change than observed for 10 mM caffeine (Wilcoxon test between caffeine and the other applied bitter substances: CAF/QUI, *p* = 0.065; CAF/DEN, *p* = 0.000; *n* = 5). Extremely weak fluorescent changes were detected in the C1 neuron labeled by *Gr22e* > *GCaMP5* in response to 10 mM caffeine (*n* = 6). Asterisks signify statistical significance (**p < 0.01).

To test whether the DP1 neuron pair shows a neuronal response to caffeine application, we monitored calcium currents in this pair of pharyngeal neurons while stimulating with caffeine. Larvae expressing *UAS-GCaMP5* in DP1 under the control of *Gr39b-GAL4* (*Gr39b* > *GCaMP5*) were dissected and introduced into a specific microfluidic device that allows controlled stimulation with taste-containing solutions (van Giesen et al., 2016). We applied 1, 10, and 100 mM caffeine and simultaneously recorded DP1 neuronal responses (**Figures 5A,B**). DP1 showed robust activity when 10 or 100 mM caffeine was applied (**Figure 5B**). Recordings of DP2 using *Gr77a* > *GCaMP5* larvae showed no neuronal response to any of the applied caffeine concentrations (**Figure 5B**). To analyze if DP1 is narrowly tuned toward caffeine, we recorded calcium-evoked fluorescence changes upon stimulation with two other bitter substances, quinine and denatonium. DP1 showed neuronal activity when stimulated with 10 mM quinine, but the response was lower than the response evoked by caffeine in the same neuron, DP1 (**Figure 5C**). No response was observed upon denatonium application (**Figure 5C**). Calcium-dependent fluorescence changes were observed in the C1 neuron when 10 mM caffeine was applied (**Figure 5C**), albeit at a significantly lower level than responses in DP1 (*p* = 0.0003). These results support that DP1 is a key player in caffeine-induced behavior.

## Discussion

In the process of feeding, the quality of food is judged at specialized external sensory organs such as the terminal organ in *Drosophila* larvae. Food is subsequently ingested through the mouth to be internalized into the digestive tract. During this process, food is exposed to the pharyngeal sense organs. Thus, it is plausible that pharyngeal sense organs provide an additional point of quality control during the feeding process, where larvae can re-evaluate whether to continue feeding on a food source, or whether to stop.

Here we identified 25 *Gr-GAL4* drivers that show expression in the pharyngeal sense organs, and constructed a detailed *Gr-GAL4* expression map of the five, five, and six neurons of the DPS, VPS, and PPS, respectively. Additionally, we observed the projection patterns of DPS GRNs to the taste center of the larval brain.

Strikingly, while examining the behavioral relevance of these pharyngeal neurons, we find that the caffeine-induced reduction in ingestion is dependent on specific pharyngeal GRNs, in particular the pair of DP1 neurons. DP1 is likely the major neuron that functions in caffeine sensing, although a possible minor involvement of other neurons cannot be completely ruled out.

The GRN organization of the pharyngeal sense organs identified in this study using *Gr-GAL4* drivers has similarities to GRN organization of the external larval head organs in certain aspects. Gr33a and Gr66a are expressed in the highest number of larval pharyngeal neurons, in 8 out of the 16 neurons that we characterized. Considering the wide-spread expression of Gr33a and Gr66a in bitter-sensing neurons (Weiss et al., 2011), at least half of the pharyngeal GRNs are likely to be involved in bitter sensing. The DP1 neuron in the DPS expresses 14 *Gr-GAL4* drivers, the highest number among the pharyngeal GRNs, comparable to the C1 neuron in the terminal organ which expresses 17 *Gr-GAL4* drivers (Kwon et al., 2011). In addition, 10 *Gr-GAL4* drivers are commonly expressed in the DP1 and C1 neurons. Based on the high number of *Gr-GAL4* drivers expressed, DP1 and C1 are likely to be the main GRNs involved in bitter sensing in larva. DP3, which expresses *Gr2a*-, *Gr23a*-, *Gr57a*-, and *Gr93d-GAL4*, and DP4 which expresses *Gr43a-GAL4*, are Gr33a- and Gr66a-independent GRNs. Similar to the CO_2_ receptors Gr21a and Gr63a in the terminal organ which show Gr33a- and Gr66a-independent expression, DP3 may be a neuron that senses a novel taste or sensory modality. Among the sugar receptors known in *Drosophila*, Gr43a is the only receptor that expresses in larva (Kwon et al., 2011; Mishra et al., 2013), and we found that it is singly expressed in DP4. We observed that *Gr93a-GAL4* is expressed in two pairs of GRNs in the DPS, DP1 and DP5, but we could not distinguish independent projection patterns (**Supplementary Figure S4**), and cannot completely rule out the possibility of non-specific leaky expression of the GAL4 driver. *Gr-GAL4* expression in the VPS and PPS shows two major characteristics. The first is that *Gr-GAL4* driver expression cannot be used to distinguish individual neurons. Most of the expressed *Gr-GAL4* drivers appear to be co-expressed. Also, when we looked at the brain projection patterns of the VPS-specific *Gr68a-GAL4* driver and PPS-specific *Gr39a.d*- and *Gr93c-GAL4* drivers, we could not distinguish single neuron projections (**Supplementary Figure S4**). In contrast, the four GRNs of the DPS showed clearly distinguishable brain projections (**Figure 2C**). These results suggest that each GRN of the DPS may be functionally distinct, while GRNs of the VPS or PPS may have similar functions within each organ. The second characteristic is that *Gr28a-GAL4* is expressed independently of Gr33a and Gr66a. *Gr28a-GAL4* expression in the external dorsal organ GRNs is also independent of Gr33a and Gr66a (Kwon et al., 2011). Thus *Gr28a-GAL4*-expressing GRNs may have novel functions, similar to DP3.

Among the chemicals we tested, 14 bitter tastants were found to cause ingestion reduction in a dose-dependent manner, and we identified the pharyngeal GRNs that detect caffeine. Several mechanisms can be proposed for how bitter chemicals other than caffeine can cause ingestion reduction. One possibility is that mechanisms or receptors other than the Grs exist to detect bitter substances and regulate ingestion. Other neurons in the pharyngeal sense organs may mediate this effect, and potential candidates could be neurons expressing members of the *ionotropic receptor* (*IR*) family or the *transient receptor potential* (*TRP*) family of cation channels. Roughly half of 35 *IR* genes in the *IR20a* clade (Koh et al., 2014) are expressed in the adult gustatory neurons. Eight genes of the *IR20a* clade are expressed in the larval pharyngeal sense organs, and seven *GAL4* drivers for these genes are expressed in the DPS (Stewart et al., 2015), consistent with a potential role in regulating ingestion. In the adult, TRPA1 (Transient receptor potential A1) is expressed in the labral sense organ (LSO) of the pharyx, and acts as a gustatory chemosensor to inhibit reactive electrophile ingestion (Kang et al., 2010). Although this and a previous study provide a comprehensive receptor-to neuron map of the Grs to 10 neurons of the DOG and TOG (Kwon et al., 2011) and 16 neurons of the pharyngeal sense organs, a majority of the ~80 gustatory neurons that exist on the external and internal organs of the larva head (Python and Stocker, 2002) are as yet uncharacterized regarding receptor expression. Expression of taste receptors such as IRs and Pickpocket (PPK) proteins has not been characterized at the cellular level and co-expression with the Grs is unknown. Examination of these and other questions in the relatively simple larval taste system should provide insight into the cellular circuits that recognize taste modalities such as sweet, bitter, and salt to result in specific behavior, as well as potential interactions between these circuits.

Our results suggest that information from the DP1 neuron is processed in a circuit that results in negative and aversive behavior in the ingestion response to caffeine. In adult *Drosophila*, Gr33a, Gr66a, and Gr93a are required for the behavioral and physiological response to caffeine (Moon et al., 2006, 2009; Lee et al., 2009), and we find that all three of these *Gr-GAL4* drivers are expressed in DP1. Since misexpression of all three of these Grs does not produce a response to caffeine (Moon et al., 2009), it is possible that other Grs expressed in DP1, or possibly other receptors such as IRs, may be essential components in caffeine recognition. DP1 not only responded to caffeine, but also responded to quinine to a certain degree in the calcium imaging experiments. Since DP1 expresses the highest number of *Gr-GAL4* drivers among the GRNs in the DPS, it is possible that DP1 responds to other bitter chemicals in addition to caffeine. However, our ingestion behavior experiments strongly suggest that DP1 is a major player in detecting caffeine to regulate ingestion, and suggest that some bitter chemicals including quinine are also detected by a mechanism independent of *Gr33a-GAL4* expressing neurons to inhibit ingestion.

In previous studies testing larval choice preference, *Gr33a*- and *Gr66a-GAL4* expressing GRNs are necessary for aversive responses to bitter substances such as caffeine, denatonium, lobeline, strychnine, and quinine (El-Keredy et al., 2012; Apostolopoulou et al., 2014; Kim et al., 2016). However, our study suggests that another mechanism other than the *Gr33a*- and *Gr66a-GAL4* expressing GRNs exists for detecting bitter tastants other than caffeine. Also in a previous study of the larval gustatory system, inactivation of GRNs of either the external terminal organ or the internal pharyngeal organs did not cause changes in choice preference, while inactivation of GRNs of both the terminal organ and pharyngeal organs impaired avoidance to bitter tastants (Kim et al., 2016). This suggested that GRNs of the terminal organ and pharyngeal organs cooperate in bitter sensing. Thus, it appears that GRNs that exist on different sensory organs may functionally cooperate or work somewhat independently in sensing different bitter substances, even in related behavioral responses such as choice preference and ingestion. Future characterization of the specific functions of each taste organ and individual GRNs should yield interesting insights.

The larval taste system has the advantages of being numerically simpler than the adult, while being amenable to various behavioral assays that measure choice preference, ingestion, post-ingestive effects, or learning. Thus, the larval taste system may provide an ideal model to comprehensively understand the workings of a whole taste system. In this study, we identified bitter tastants that affect larval ingestion behavior, and found that specific pharyngeal GRNs play a major role in caffeine-mediated ingestion reduction, and thus provide a piece of the puzzle of the *Drosophila* larval taste system.

## Author Contributions

JC, SS, and JK designed research. JC and LG performed research. JC, LG, MC, KK, SS, and JK analyzed data. JC, LG, MC, SS, and JK wrote the manuscript. All authors read and approved the final manuscript.

## Conflict of Interest Statement

The authors declare that the research was conducted in the absence of any commercial or financial relationships that could be construed as a potential conflict of interest.

## Supplementary Material

FIGURE S1 **Amounts of indigo carmine are proportional to OD values. (A)** OD values of indigo carmine concentrations of 0.001–10 M on a log scale. **(B)** For the OD value range of 0.1–0.4 [marked as a dotted box in **(A)**], which was the range measured in our ingestion assay, the OD value appears to be linearly proportional to the amount of indigo carmine dye.

FIGURE S2 **Double labeling of *Gr33a-QF* and *GrX-GAL4* in the pharyngeal sense organs. (A–C)** Examination of whether *Gr-GAL4* drivers that express in the DPS **(A)**, VPS **(B)**, and PPS **(C)** co-express with *Gr33a-QF*. Red is expression from *Gr33a-QF*> *QUAS-mtdTomato*, and green is expression from *GrX-GAL4* > *UAS-mcd8-GFP*. The photos are taken at the positions shown as boxes in **Figure 1A**. Arrowheads indicate *Gr-GAL4* expressing neurons that are independent of *Gr33a-QF* expression.

FIGURE S3 **Influence of smell on ingestion behavior of *Drosophila* larva**. Comparison of ingestion of *wCS* and *orco^1^* larvae to the indicated bitter tastants at various concentrations. For each data, 6 < *n* < 18. **p* < 0.05, ***p* < 0.01, *Mann–Whitney U* test pair-wise comparisons. The *p*-values of the points marked with asterisks are as follows: 10 mM LOB, *p* = 0.041; 5 mM QUI, *p* = 0.018; 1 mM COU, *p* = 0.013; 10 mM COU, *p* = 0.000; 0.1 mM ESC, *p* = 0.002.

FIGURE S4 **Projection patterns of pharyngeal GRNs in the larval brain. (A)** Projection pattern of *Gr93a-GAL4*, which is expressed in DP1 and DP5. The arrowheads indicate expression from the odorant receptor neurons in the dorsal organ projecting to the larval antennal lobe, and thus is likely to be ectopic expression. **(B)** Projection patterns of Gr-GAL4 drivers that only express in either the VPS or PPS, and a schematic projection map based on these patterns. The brain neuropil is counterstained with the monoclonal antibody nc82 (magenta).

TABLE S1 **Average number of *Gr-GAL4*-driven GFP-labeled cell pairs in the pharyngeal sense organs**. Each *Gr-GAL4* line was quantified for the number of neurons expressing the GFP reporter in the pharyngeal sense organs (DPS, VPS, and PPS). For the combinations of each *Gr-GAL4* line and *Gr2a-GAL4*, the numbers of GFP-expressing neurons in the DPS were also quantified. When the number of neurons was additive upon combination of two drivers, the drivers were assumed to express in independent neurons.

TABLE S2 **Ingestion assay results for 22 putative bitter tastants**. Actual measurements used to construct the graphs in **Figure 3** and calculations of the ingestion index (I.I.) are provided. The abbreviations for each chemical are noted, and the order of the chemicals is the same as **Figure 3**.
