# Supplementary figures and images for: A Pair of Pharyngeal Gustatory Receptor Neurons Regulates Caffeine-Dependent Ingestion in Drosophila Larvae
Source: Front Cell Neurosci. 2016 Jul 19;10:181. doi: 10.3389/fncel.2016.00181 (PMC4949222; doi:10.3389/fncel.2016.00181)

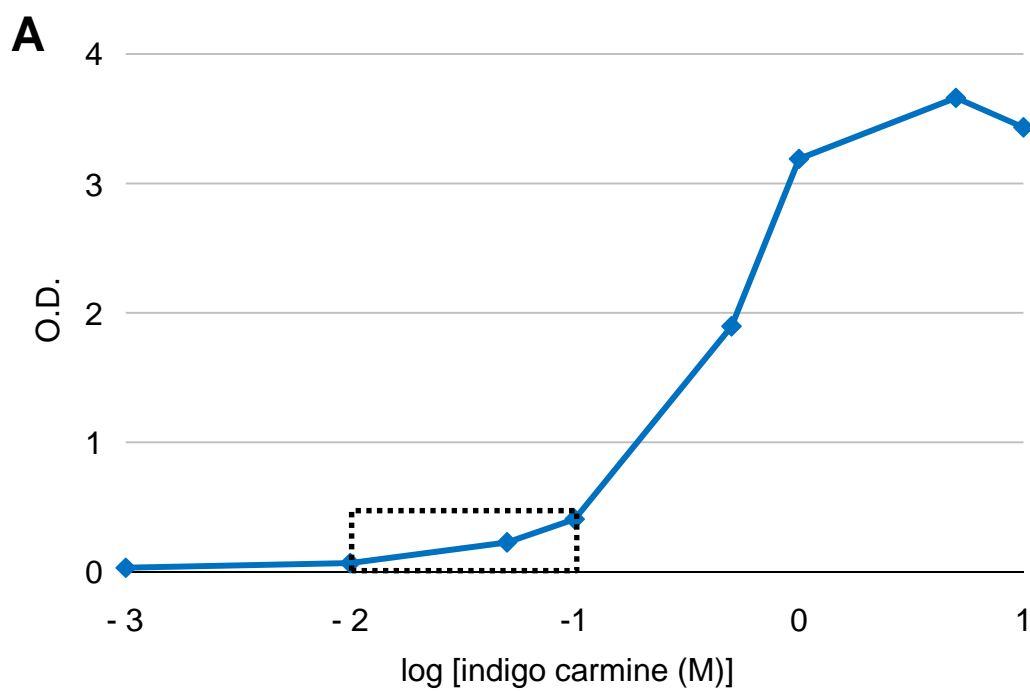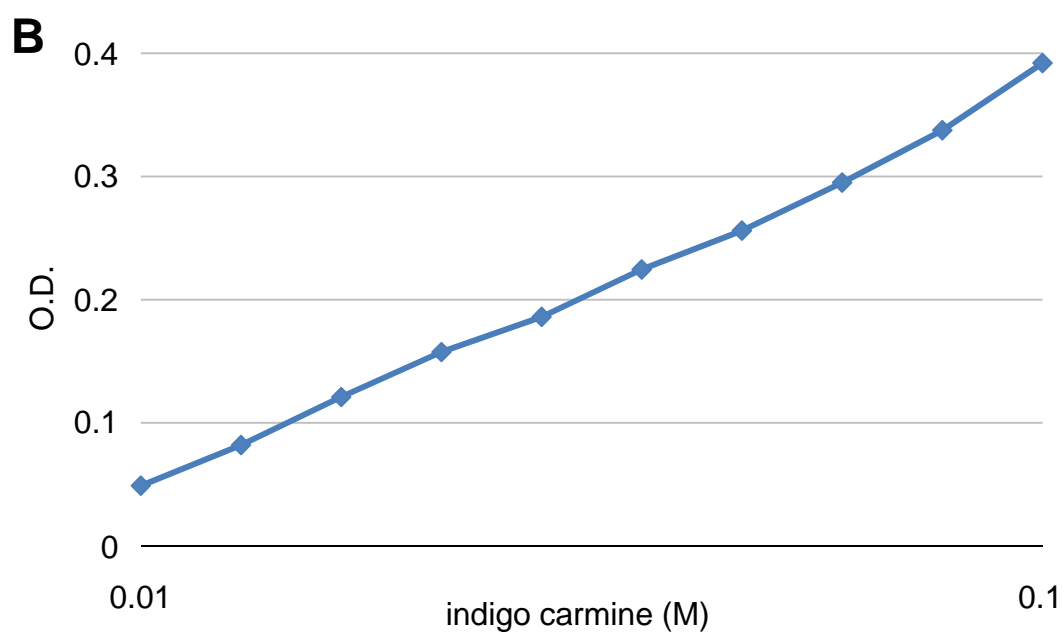

Supplement: FIGURE S1 — Amounts of indigo carmine are proportional to OD values. (A) OD values of indigo carmine concentrations of 0.001–10 M on a log scale. (B) For the OD value range of 0.1–0.4 [marked as a dotted box in (A)], which was the range measured in our ingestion assay, the OD value appears to be linearly proportional to the amount of indigo carmine dye. [file Image_1.PDF]

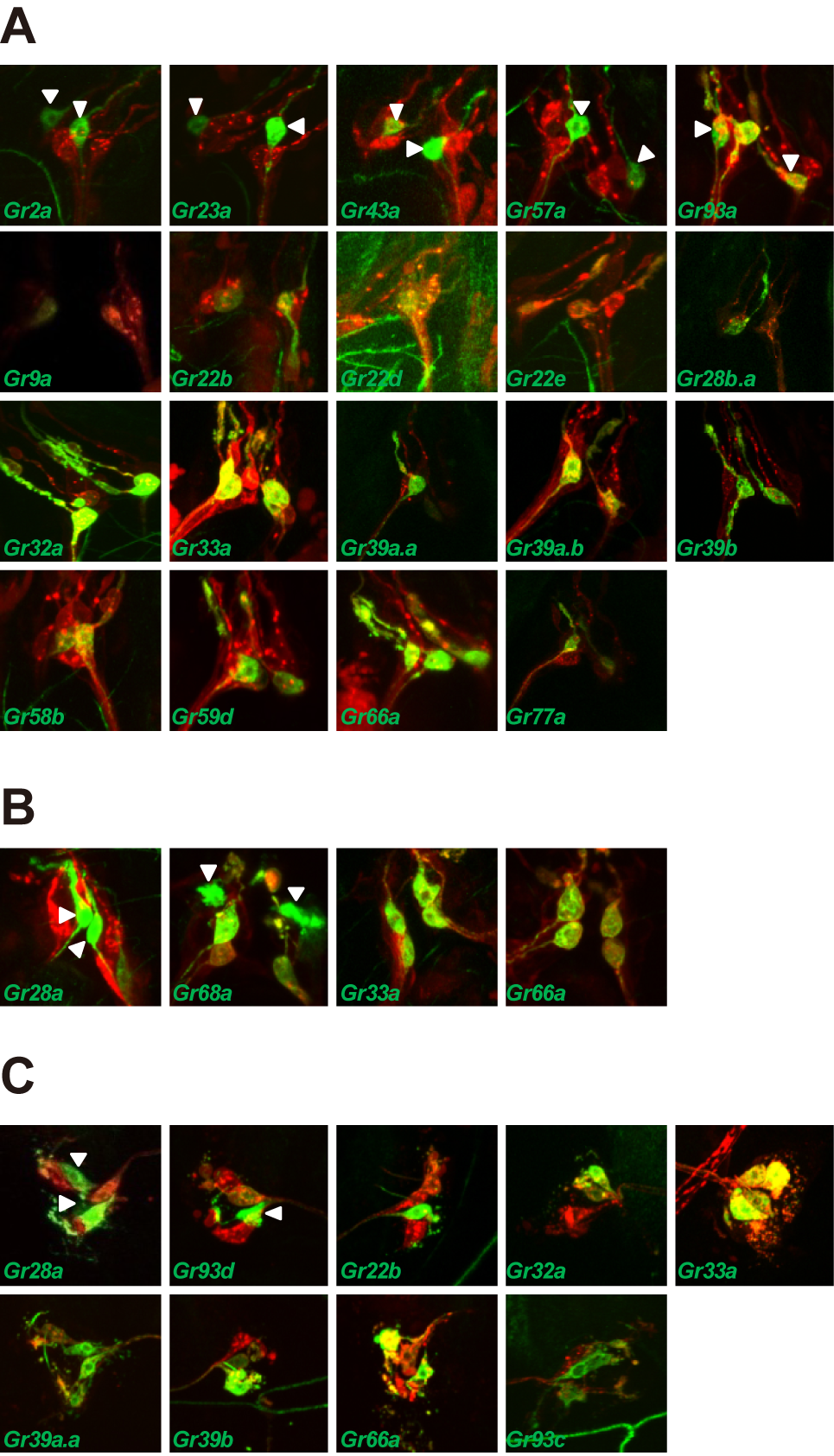

Supplement: FIGURE S2 — Double labeling of Gr33a-QF and GrX-GAL4 in the pharyngeal sense organs. (A–C) Examination of whether Gr-GAL4 drivers that express in the DPS (A), VPS (B), and PPS (C) co-express with Gr33a-QF. Red is expression from Gr33a-QF> QUAS-mtdTomato, and green is expression from GrX-GAL4 > UAS-mcd8-GFP. The photos are taken at the positions shown as boxes in Figure 1A. Arrowheads indicate Gr-GAL4 expressing neurons that are independent of Gr33a-QF expression. [file Image_2.TIF]

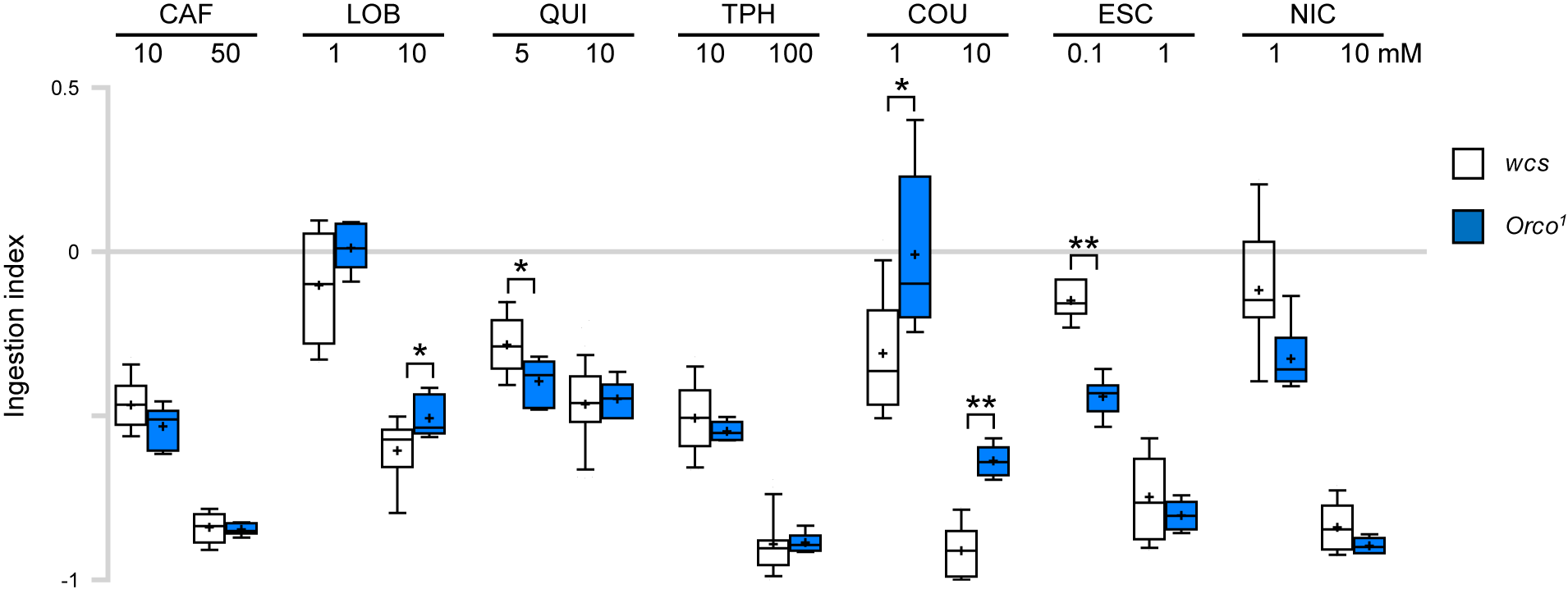

Supplement: FIGURE S3 — Influence of smell on ingestion behavior of Drosophila larva. Comparison of ingestion of wCS and orco1 larvae to the indicated bitter tastants at various concentrations. For each data, 6 < n < 18. *p < 0.05, **p < 0.01, Mann–Whitney U test pair-wise comparisons. The p-values of the points marked with asterisks are as follows: 10 mM LOB, p = 0.041; 5 mM QUI, p = 0.018; 1 mM COU, p = 0.013; 10 mM COU, p = 0.000; 0.1 mM ESC, p = 0.002. [file Image_3.TIF]

**A**

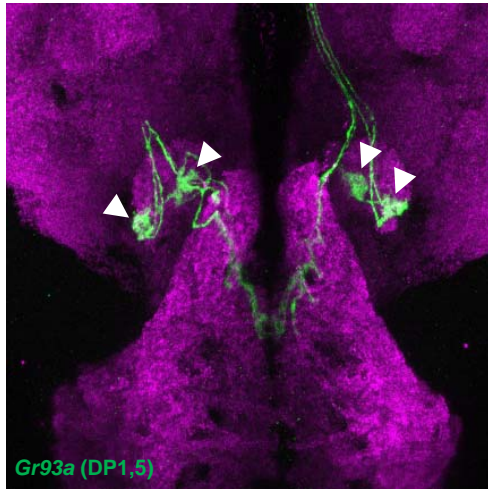

**B**

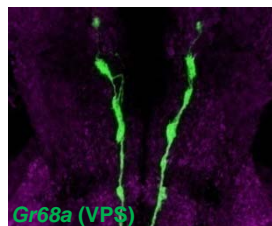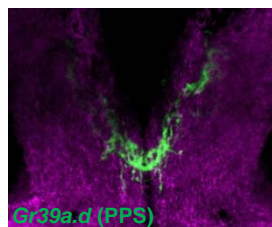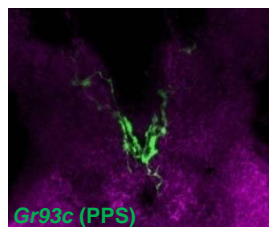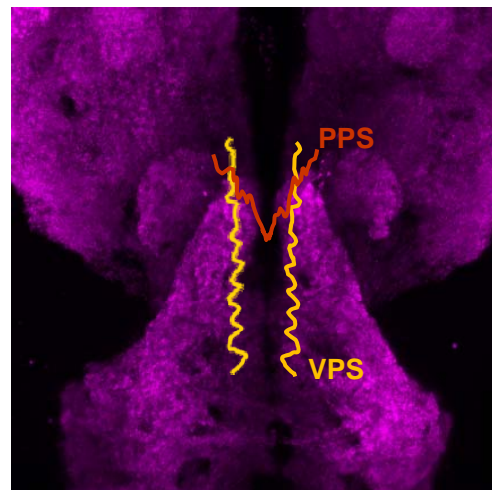

Supplement: FIGURE S4 — Projection patterns of pharyngeal GRNs in the larval brain. (A) Projection pattern of Gr93a-GAL4, which is expressed in DP1 and DP5. The arrowheads indicate expression from the odorant receptor neurons in the dorsal organ projecting to the larval antennal lobe, and thus is likely to be ectopic expression. (B) Projection patterns of Gr-GAL4 drivers that only express in either the VPS or PPS, and a schematic projection map based on these patterns. The brain neuropil is counterstained with the monoclonal antibody nc82 (magenta). [file Image_4.PDF]
