# Supplementary material for: A Pair of Pharyngeal Gustatory Receptor Neurons Regulates Caffeine-Dependent Ingestion in Drosophila Larvae
Source: Front Cell Neurosci. 2016 Jul 19;10:181. doi: 10.3389/fncel.2016.00181 (PMC4949222; doi:10.3389/fncel.2016.00181)
Supplement: TABLE S1 — Average number of Gr-GAL4-driven GFP-labeled cell pairs in the pharyngeal sense organs. Each Gr-GAL4 line was quantified for the number of neurons expressing the GFP reporter in the pharyngeal sense organs (DPS, VPS, and PPS). For the combinations of each Gr-GAL4 line and Gr2a-GAL4, the numbers of GFP-expressing neurons in the DPS were also quantified. When the number of neurons was additive upon combination of two drivers, the drivers were assumed to express in independent neurons. [file Table_1.PDF]

	Pharyngeal organs			X *Gr2a-GAL4*
	DPS	VPS	PPS	DPS
Gr2a	1.00	·	·	1.00
Gr9a	0.71	·	·	1.28
Gr22b	1.00	·	3.06	1.95
Gr22d	0.75	·	·	1.78
Gr22e	1.00	·	·	1.96
Gr23a	1.00	·	·	0.97
Gr28a	·	2.27	2.32	·
Gr28b.a	0.95	·	·	1.38
Gr32a	1.78	·	2.40	2.15
Gr33a	1.75	1.88	2.07	2.30
Gr39a.a	0.80	·	2.80	1.90
Gr39a.b	0.98	·	·	1.83
Gr39a.d	·	·	1.80	·
Gr39b	1.00	·	0.49	1.96
Gr43a	0.98	·	·	1.99
Gr57a	0.90	·	·	1.00
Gr58b	0.92	·	·	1.98
Gr59d	1.00	·	·	1.99
Gr66a	1.03	1.93	0.42	2.05
Gr68a	·	2.98	·	·
Gr77a	0.93	·	·	1.86
Gr93a	1.03	·	·	1.95
Gr93b	0.98	·	·	1.13
Gr93c	·	·	2.03	·
Gr93d	0.94	·	1.88	1.03
