# Supplementary material for: A Pair of Pharyngeal Gustatory Receptor Neurons Regulates Caffeine-Dependent Ingestion in Drosophila Larvae
Source: Front Cell Neurosci. 2016 Jul 19;10:181. doi: 10.3389/fncel.2016.00181 (PMC4949222; doi:10.3389/fncel.2016.00181)
Supplement: TABLE S2 — Ingestion assay results for 22 putative bitter tastants. Actual measurements used to construct the graphs in Figure 3 and calculations of the ingestion index (I.I.) are provided. The abbreviations for each chemical are noted, and the order of the chemicals is the same as Figure 3. [file Table_2.PDF]

Chemical (abbreviation)	concentration (mM)	experimental O.D. – empty O.D.	dye only control O.D. – empty O.D.	I.I. = ((experimental O.D. – empty O.D.) – (dye only control O.D. – empty O.D.)) / (dye only control O.D. – empty O.D.)
atropine (ATR)	0.1	0.414	0.367	0.128
	0.1	0.311	0.311	0.000
	0.1	0.418	0.341	0.226
	0.1	0.325	0.326	-0.003
	0.1	0.331	0.341	-0.029
	0.1	0.367	0.342	0.073
	1	0.241	0.314	-0.232
	1	0.217	0.308	-0.295
	1	0.216	0.291	-0.258
	1	0.172	0.211	-0.185
	1	0.156	0.256	-0.391
	1	0.176	0.169	0.041
	10	0.229	0.314	-0.271
	10	0.204	0.308	-0.338
	10	0.216	0.291	-0.258
	10	0.132	0.211	-0.374
	10	0.094	0.256	-0.633
	10	0.122	0.169	-0.278
berberine chloride (BER)	0.1	0.224	0.222	0.009
	0.1	0.197	0.200	-0.015
	0.1	0.245	0.329	-0.255
	0.1	0.177	0.314	-0.436
	0.1	0.211	0.182	0.159
	0.1	0.227	0.200	0.135
	1	0.179	0.222	-0.194
	1	0.175	0.200	-0.125
	1	0.294	0.329	-0.106
	1	0.231	0.314	-0.264
	1	0.179	0.182	-0.016
	1	0.198	0.200	-0.010
	5	0.191	0.275	-0.305
	5	0.207	0.327	-0.367
	5	0.172	0.307	-0.440
	5	0.178	0.301	-0.409
	5	0.183	0.316	-0.421
	5	0.193	0.296	-0.348
	5	0.293	0.396	-0.260
caffeine (CAF)	1	0.302	0.281	0.075
	1	0.284	0.254	0.118
	1	0.232	0.220	0.055
	1	0.297	0.285	0.042
	1	0.298	0.293	0.017
	1	0.325	0.307	0.059
	1	0.249	0.262	-0.050
	1	0.230	0.274	-0.161
	1	0.288	0.291	-0.010
	1	0.279	0.267	0.045
	1	0.224	0.244	-0.082

caffeine (CAF)	1	0.219	0.290	-0.245
	10	0.154	0.281	-0.452
	10	0.153	0.254	-0.398
	10	0.123	0.220	-0.441
	10	0.173	0.285	-0.393
	10	0.145	0.293	-0.505
	10	0.140	0.307	-0.544
	10	0.178	0.262	-0.321
	10	0.129	0.274	-0.529
	10	0.139	0.291	-0.522
	10	0.148	0.267	-0.446
	10	0.105	0.244	-0.570
	10	0.151	0.290	-0.479
	100	0.032	0.341	-0.906
	100	0.024	0.309	-0.922
	100	0.025	0.274	-0.909
	100	0.032	0.339	-0.906
	100	0.035	0.338	-0.896
	100	0.026	0.364	-0.929
coumarin (COU)	0.1	0.337	0.367	-0.082
	0.1	0.321	0.311	0.032
	0.1	0.389	0.341	0.141
	0.1	0.319	0.326	-0.021
	0.1	0.332	0.341	-0.026
	0.1	0.340	0.342	-0.006
	1	0.129	0.264	-0.511
	1	0.141	0.228	-0.382
	1	0.111	0.221	-0.498
	1	0.147	0.258	-0.430
	1	0.122	0.199	-0.387
	1	0.124	0.237	-0.477
	1	0.196	0.241	-0.187
	1	0.254	0.245	0.037
	1	0.170	0.205	-0.171
	1	0.221	0.268	-0.175
	1	0.199	0.247	-0.194
	1	0.164	0.250	-0.344
	10	0.001	0.264	-0.996
	10	0.007	0.228	-0.969
	10	0.007	0.221	-0.968
	10	0.016	0.258	-0.938
	10	0.000	0.199	-1.000
	10	0.000	0.237	-1.000
	10	0.053	0.241	-0.780
	10	0.049	0.245	-0.800
	10	0.032	0.205	-0.844
	10	0.031	0.268	-0.884
	10	0.032	0.247	-0.870
	10	0.031	0.250	-0.876
escin (ESC)	0.01	0.201	0.305	-0.341
	0.01	0.207	0.311	-0.334
	0.01	0.212	0.181	0.171
	0.01	0.178	0.164	0.085

escin (ESC)	0.01	0.234	0.177	0.322
	0.01	0.195	0.179	0.089
	0.1	0.157	0.305	-0.485
	0.1	0.123	0.311	-0.605
	0.1	0.136	0.181	-0.249
	0.1	0.121	0.164	-0.262
	0.1	0.192	0.177	0.085
	0.1	0.151	0.179	-0.156
	0.1	0.242	0.293	-0.174
	0.1	0.271	0.296	-0.084
	0.1	0.242	0.292	-0.171
	0.1	0.241	0.263	-0.084
	0.1	0.237	0.308	-0.231
	0.1	0.290	0.338	-0.142
	1	0.040	0.218	-0.817
	1	0.040	0.204	-0.804
	1	0.025	0.220	-0.886
	1	0.030	0.214	-0.860
	1	0.020	0.218	-0.908
	1	0.028	0.235	-0.881
	1	0.133	0.293	-0.546
	1	0.110	0.296	-0.628
	1	0.105	0.292	-0.640
	1	0.100	0.263	-0.620
	1	0.110	0.308	-0.643
	1	0.093	0.338	-0.725
(-)-lobeline hydrochloride (LOB)	0.1	0.232	0.182	0.275
	0.1	0.215	0.242	-0.112
	0.1	0.229	0.234	-0.021
	0.1	0.207	0.239	-0.134
	0.1	0.239	0.238	0.004
	0.1	0.296	0.258	0.147
	0.1	0.298	0.282	0.057
	0.1	0.328	0.278	0.180
	0.1	0.263	0.344	-0.235
	0.1	0.278	0.301	-0.076
	0.1	0.345	0.303	0.139
	0.1	0.317	0.328	-0.034
	1	0.199	0.182	0.093
	1	0.198	0.242	-0.182
	1	0.212	0.234	-0.094
	1	0.159	0.239	-0.335
	1	0.164	0.238	-0.311
	1	0.253	0.258	-0.019
	1	0.309	0.282	0.096
	1	0.298	0.278	0.072
	1	0.309	0.344	-0.102
	1	0.261	0.301	-0.133
	1	0.305	0.303	0.007
	1	0.225	0.328	-0.314
	10	0.077	0.182	-0.577
	10	0.081	0.242	-0.665
	10	0.101	0.234	-0.568

(-)-lobeline hydrochloride (LOB)	10	0.049	0.239	-0.795
	10	0.098	0.238	-0.588
	10	0.053	0.258	-0.795
	10	0.135	0.282	-0.521
	10	0.141	0.278	-0.493
	10	0.157	0.344	-0.544
	10	0.134	0.301	-0.555
	10	0.139	0.303	-0.541
	10	0.122	0.328	-0.628
(-)-nicotine (NIC)	0.1	0.229	0.317	-0.278
	0.1	0.239	0.341	-0.299
	0.1	0.221	0.220	0.005
	0.1	0.203	0.212	-0.042
	0.1	0.205	0.232	-0.116
	0.1	0.251	0.225	0.116
	0.1	0.501	0.415	0.207
	0.1	0.439	0.361	0.216
	0.1	0.489	0.375	0.304
	0.1	0.524	0.372	0.409
	0.1	0.498	0.431	0.155
	0.1	0.516	0.409	0.262
	1	0.193	0.317	-0.391
	1	0.206	0.341	-0.396
	1	0.188	0.220	-0.145
	1	0.181	0.212	-0.146
	1	0.190	0.232	-0.181
	1	0.192	0.225	-0.147
	1	0.386	0.415	-0.070
	1	0.384	0.361	0.064
	1	0.299	0.375	-0.203
	1	0.436	0.372	0.172
	1	0.526	0.431	0.220
	1	0.333	0.409	-0.186
	10	0.029	0.317	-0.909
	10	0.025	0.341	-0.927
	10	0.023	0.220	-0.895
	10	0.018	0.212	-0.915
	10	0.031	0.232	-0.866
	10	0.022	0.225	-0.902
	10	0.094	0.415	-0.773
	10	0.082	0.361	-0.773
	10	0.089	0.375	-0.763
	10	0.107	0.372	-0.712
	10	0.078	0.431	-0.819
	10	0.071	0.409	-0.826
N-phenylthiourea (PTU)	0.01	0.379	0.335	0.131
	0.01	0.378	0.298	0.268
	0.01	0.355	0.311	0.141
	0.01	0.345	0.301	0.146
	0.01	0.357	0.323	0.105
	0.01	0.447	0.484	-0.076
	0.01	0.210	0.264	-0.205
	0.01	0.164	0.248	-0.339

N-phenylthiourea (PTU)	0.01	0.185	0.198	-0.066
	0.01	0.169	0.195	-0.133
	0.01	0.155	0.171	-0.094
	0.1	0.362	0.335	0.081
	0.1	0.372	0.298	0.248
	0.1	0.376	0.311	0.209
	0.1	0.382	0.301	0.269
	0.1	0.277	0.323	-0.142
	0.1	0.414	0.484	-0.145
	1	0.188	0.264	-0.288
	1	0.175	0.248	-0.294
	1	0.181	0.198	-0.086
	1	0.154	0.195	-0.210
	1	0.128	0.171	-0.251
quinine hydrochloride dihydrate (QUI)	0.1	0.283	0.306	-0.075
	0.1	0.319	0.297	0.074
	0.1	0.364	0.391	-0.069
	0.1	0.293	0.299	-0.020
	0.1	0.338	0.359	-0.058
	0.1	0.340	0.369	-0.079
	1	0.168	0.260	-0.354
	1	0.261	0.245	0.065
	1	0.229	0.229	0.000
	1	0.176	0.226	-0.221
	1	0.182	0.239	-0.238
	1	0.239	0.268	-0.108
	1	0.231	0.257	-0.101
	1	0.236	0.261	-0.096
	1	0.239	0.273	-0.125
	1	0.202	0.236	-0.144
	1	0.195	0.263	-0.259
	1	0.205	0.183	0.120
	1	0.161	0.201	-0.199
	1	0.200	0.221	-0.095
	1	0.165	0.171	-0.035
	1	0.180	0.200	-0.100
	1	0.197	0.194	0.015
	1	0.196	0.240	-0.183
	1	0.202	0.166	0.217
	1	0.207	0.201	0.030
	1	0.184	0.183	0.005
	1	0.171	0.181	-0.055
	1	0.179	0.203	-0.118
	1	0.202	0.191	0.058
	10	0.082	0.260	-0.685
	10	0.076	0.245	-0.690
	10	0.089	0.229	-0.611
	10	0.071	0.226	-0.686
	10	0.086	0.239	-0.640
	10	0.093	0.268	-0.653
	10	0.153	0.257	-0.405
	10	0.151	0.261	-0.421
	10	0.150	0.273	-0.451

quinine hydrochloride dihydrate (QUI)	10	0.125	0.236	-0.470
	10	0.129	0.263	-0.510
	10	0.137	0.240	-0.429
	10	0.145	0.183	-0.208
	10	0.102	0.201	-0.493
	10	0.110	0.221	-0.502
	10	0.122	0.171	-0.287
	10	0.100	0.200	-0.500
	10	0.111	0.194	-0.428
	10	0.132	0.215	-0.386
	10	0.114	0.177	-0.356
	10	0.105	0.227	-0.537
	10	0.106	0.221	-0.520
	10	0.105	0.214	-0.509
	10	0.129	0.207	-0.377
	10	0.114	0.232	-0.509
	10	0.109	0.224	-0.513
	10	0.105	0.257	-0.591
	10	0.125	0.187	-0.332
	10	0.128	0.221	-0.421
	10	0.121	0.214	-0.435
	10	0.088	0.166	-0.470
	10	0.117	0.201	-0.418
	10	0.114	0.183	-0.377
	10	0.122	0.181	-0.326
	10	0.130	0.203	-0.360
	10	0.147	0.191	-0.230
D-(+)-sucrose octaacetate (SOA)	0.01	0.320	0.320	0.000
	0.01	0.329	0.392	-0.161
	0.01	0.336	0.392	-0.143
	0.01	0.399	0.421	-0.052
	0.01	0.372	0.369	0.008
	0.01	0.371	0.347	0.069
	0.1	0.149	0.255	-0.416
	0.1	0.178	0.231	-0.229
	0.1	0.155	0.213	-0.272
	0.1	0.161	0.167	-0.036
	0.1	0.150	0.177	-0.153
	0.1	0.176	0.179	-0.017
	1	0.124	0.255	-0.514
	1	0.183	0.231	-0.208
	1	0.169	0.213	-0.207
	1	0.114	0.167	-0.317
	1	0.137	0.177	-0.226
	1	0.130	0.179	-0.274
strychnine nitrate (STR)	0.1	0.349	0.320	0.091
	0.1	0.340	0.392	-0.133
	0.1	0.357	0.392	-0.089
	0.1	0.383	0.421	-0.090
	0.1	0.303	0.369	-0.179
	0.1	0.345	0.347	-0.006
	1	0.184	0.303	-0.393
	1	0.179	0.268	-0.332

strychnine nitrate (STR)	1	0.178	0.236	-0.246
	1	0.179	0.234	-0.235
	1	0.161	0.223	-0.278
	1	0.181	0.251	-0.279
	10	0.115	0.303	-0.620
	10	0.117	0.268	-0.563
	10	0.087	0.236	-0.631
	10	0.055	0.234	-0.765
	10	0.053	0.223	-0.762
	10	0.075	0.251	-0.701
theophylline anhydrous (TPH)	1	0.256	0.234	0.094
	1	0.242	0.277	-0.126
	1	0.223	0.251	-0.112
	1	0.211	0.246	-0.142
	1	0.240	0.242	-0.008
	1	0.286	0.274	0.044
	1	0.253	0.244	0.037
	1	0.404	0.270	0.496
	1	0.246	0.473	-0.480
	1	0.240	0.251	-0.044
	1	0.212	0.249	-0.149
	1	0.254	0.254	0.000
	1	0.207	0.238	-0.130
	1	0.228	0.227	0.004
	1	0.199	0.254	-0.217
	1	0.208	0.244	-0.148
	1	0.247	0.260	-0.050
	1	0.168	0.146	0.151
	10	0.119	0.234	-0.491
	10	0.132	0.277	-0.523
	10	0.106	0.251	-0.578
	10	0.124	0.246	-0.496
	10	0.134	0.242	-0.446
	10	0.122	0.274	-0.555
	10	0.156	0.244	-0.361
	10	0.184	0.270	-0.319
	10	0.207	0.473	-0.562
	10	0.164	0.251	-0.347
	10	0.123	0.249	-0.506
	10	0.160	0.254	-0.370
	10	0.098	0.238	-0.588
	10	0.092	0.227	-0.595
	10	0.086	0.254	-0.661
	10	0.082	0.244	-0.664
	10	0.094	0.260	-0.638
	10	0.076	0.146	-0.479
	10	0.099	0.170	-0.418
	10	0.072	0.189	-0.619
	10	0.093	0.162	-0.426
	100	0.004	0.302	-0.987
	100	0.003	0.304	-0.990
	100	0.000	0.274	-1.000
	100	0.006	0.365	-0.984

theophylline anhydrous (TPH)	100	0.006	0.271	-0.978
	100	0.013	0.286	-0.955
	100	0.069	0.244	-0.717
	100	0.069	0.270	-0.744
	100	0.065	0.473	-0.863
	100	0.077	0.251	-0.693
	100	0.062	0.249	-0.751
	100	0.061	0.254	-0.760
	100	0.025	0.238	-0.895
	100	0.026	0.227	-0.885
	100	0.026	0.254	-0.898
	100	0.012	0.244	-0.951
	100	0.018	0.260	-0.931
	100	0.016	0.146	-0.890
	100	0.028	0.232	-0.879
	100	0.021	0.224	-0.906
	100	0.025	0.257	-0.903
	100	0.020	0.187	-0.893
	100	0.019	0.221	-0.914
	100	0.018	0.214	-0.916
	100	0.019	0.170	-0.888
	100	0.008	0.189	-0.958
	100	0.014	0.162	-0.914
umbelliferone (UMB)	0.1	0.321	0.306	0.049
	0.1	0.289	0.297	-0.027
	0.1	0.291	0.391	-0.256
	0.1	0.327	0.299	0.094
	0.1	0.352	0.359	-0.019
	0.1	0.372	0.369	0.008
	1	0.172	0.240	-0.283
	1	0.179	0.217	-0.175
	1	0.158	0.174	-0.092
	1	0.157	0.171	-0.082
	1	0.192	0.236	-0.186
	1	0.166	0.210	-0.210
	10	0.108	0.240	-0.550
	10	0.150	0.217	-0.309
	10	0.104	0.174	-0.402
	10	0.119	0.171	-0.304
	10	0.138	0.236	-0.415
	10	0.124	0.210	-0.410
N,N-diethyl-m-toluamide (DEET)	0.01%	0.165	0.268	-0.384
	0.01%	0.182	0.166	0.096
	0.01%	0.172	0.205	-0.161
	0.01%	0.160	0.186	-0.140
	0.01%	0.164	0.249	-0.341
	0.01%	0.174	0.229	-0.240
	0.1%	0.111	0.268	-0.586
	0.1%	0.122	0.166	-0.265
	0.1%	0.121	0.205	-0.410
	0.1%	0.109	0.186	-0.414
	0.1%	0.138	0.249	-0.446
	0.1%	0.103	0.229	-0.550

N,N-diethyl-m-toluamide (DEET)	1%	0.010	0.226	-0.956
	1%	0.002	0.244	-0.992
	1%	0.001	0.245	-0.996
	1%	0.000	0.220	-1.000
	1%	0.009	0.250	-0.964
	1%	0.009	0.287	-0.969
	1%	0.027	0.268	-0.899
	1%	0.029	0.166	-0.825
	1%	0.018	0.205	-0.912
	1%	0.018	0.186	-0.903
	1%	0.008	0.249	-0.968
	1%	0.002	0.229	-0.991
(+)-catechin (CAT)	0.1	0.368	0.348	0.057
	0.1	0.411	0.371	0.108
	0.1	0.357	0.359	-0.006
	0.1	0.338	0.331	0.021
	0.1	0.432	0.352	0.227
	0.1	0.373	0.347	0.075
	1	0.261	0.315	-0.171
	1	0.306	0.266	0.150
	1	0.306	0.290	0.055
	1	0.311	0.369	-0.157
	1	0.272	0.234	0.162
	1	0.344	0.367	-0.063
	10	0.300	0.315	-0.048
	10	0.317	0.266	0.192
	10	0.300	0.290	0.034
	10	0.289	0.369	-0.217
	10	0.291	0.234	0.244
	10	0.314	0.367	-0.144
denatonium benzoate (DEN)	0.1	0.218	0.169	0.290
	0.1	0.188	0.175	0.074
	0.1	0.176	0.185	-0.049
	0.1	0.215	0.169	0.272
	0.1	0.211	0.175	0.206
	0.1	0.233	0.206	0.131
	0.1	0.296	0.327	-0.095
	0.1	0.352	0.332	0.060
	0.1	0.304	0.378	-0.196
	0.1	0.351	0.295	0.190
	0.1	0.349	0.375	-0.069
	0.1	0.317	0.313	0.013
	1	0.180	0.169	0.065
	1	0.167	0.175	-0.046
	1	0.175	0.185	-0.054
	1	0.173	0.169	0.024
	1	0.195	0.175	0.114
	1	0.203	0.206	-0.015
	1	0.246	0.250	-0.016
	1	0.276	0.262	0.053
	1	0.349	0.275	0.269
	1	0.298	0.297	0.003
	1	0.306	0.235	0.302

denatonium benzoate (DEN)	1	0.213	0.253	-0.158
	10	0.131	0.169	-0.225
	10	0.177	0.175	0.011
	10	0.102	0.185	-0.449
	10	0.107	0.169	-0.367
	10	0.080	0.175	-0.543
	10	0.129	0.206	-0.374
	10	0.275	0.250	0.100
	10	0.295	0.262	0.126
	10	0.299	0.275	0.087
	10	0.314	0.297	0.057
	10	0.291	0.235	0.238
	10	0.251	0.253	-0.008
	10	0.275	0.214	0.285
	10	0.261	0.265	-0.015
	10	0.251	0.202	0.243
	10	0.197	0.236	-0.165
	10	0.215	0.251	-0.143
	10	0.253	0.263	-0.038
harmaline (HAR)	0.1	0.161	0.236	-0.318
	0.1	0.161	0.253	-0.364
	0.1	0.173	0.167	0.036
	0.1	0.158	0.181	-0.127
	0.1	0.189	0.181	0.044
	0.1	0.154	0.129	0.194
	0.1	0.292	0.289	0.010
	0.1	0.321	0.309	0.039
	0.1	0.354	0.273	0.297
	0.1	0.354	0.318	0.113
	0.1	0.331	0.317	0.044
	0.1	0.322	0.304	0.059
	0.1	0.252	0.299	-0.157
	0.1	0.238	0.236	0.008
	0.1	0.279	0.304	-0.082
	0.1	0.190	0.326	-0.417
	0.1	0.258	0.276	-0.065
	0.1	0.215	0.270	-0.204
	1	0.258	0.236	0.093
	1	0.147	0.253	-0.419
	1	0.216	0.167	0.293
	1	0.165	0.181	-0.088
	1	0.207	0.181	0.144
	1	0.158	0.129	0.225
	1	0.254	0.299	-0.151
	1	0.230	0.236	-0.025
	1	0.264	0.304	-0.132
	1	0.218	0.326	-0.331
	1	0.308	0.276	0.116
	1	0.276	0.270	0.022
	5	0.148	0.154	-0.039
	5	0.127	0.130	-0.023
	5	0.135	0.144	-0.062
	5	0.169	0.163	0.037

harmaline (HAR)	5	0.150	0.134	0.119
	5	0.115	0.115	0.000
saponin (SAP)	0.001%	0.384	0.441	-0.129
	0.001%	0.415	0.385	0.078
	0.001%	0.390	0.396	-0.015
	0.001%	0.443	0.396	0.119
	0.001%	0.436	0.439	-0.007
	0.001%	0.384	0.387	-0.008
	0.01%	0.460	0.441	0.043
	0.01%	0.433	0.385	0.125
	0.01%	0.466	0.396	0.177
	0.01%	0.465	0.396	0.174
	0.01%	0.486	0.439	0.107
	0.01%	0.391	0.387	0.010
	0.01%	0.331	0.379	-0.127
	0.01%	0.345	0.389	-0.113
	0.01%	0.206	0.261	-0.211
	0.01%	0.200	0.246	-0.187
	0.01%	0.193	0.206	-0.063
	0.01%	0.290	0.334	-0.132
	0.1%	0.385	0.441	-0.127
	0.1%	0.429	0.385	0.114
	0.1%	0.444	0.396	0.121
	0.1%	0.386	0.396	-0.025
	0.1%	0.422	0.439	-0.039
	0.1%	0.386	0.387	-0.003
tannic acid (TAA)	0.01	0.317	0.334	-0.051
	0.01	0.216	0.584	-0.630
	0.01	0.150	0.279	-0.462
	0.01	0.299	0.423	-0.293
	0.01	0.173	0.203	-0.148
	0.01	0.328	0.279	0.176
	0.01	0.321	0.269	0.193
	0.01	0.330	0.265	0.245
	0.01	0.358	0.257	0.393
	0.01	0.369	0.275	0.342
	0.01	0.305	0.276	0.105
	0.01	0.253	0.282	-0.103
	0.01	0.126	0.154	-0.182
	0.01	0.174	0.149	0.168
	0.01	0.110	0.137	-0.197
	0.01	0.126	0.134	-0.060
	0.01	0.175	0.152	0.151
	0.01	0.200	0.126	0.587
	0.1	0.332	0.334	-0.006
	0.1	0.250	0.584	-0.572
	0.1	0.253	0.279	-0.093
	0.1	0.352	0.423	-0.168
	0.1	0.318	0.203	0.567
	0.1	0.414	0.279	0.484
	0.1	0.266	0.269	-0.011
	0.1	0.283	0.265	0.068
	0.1	0.299	0.257	0.163

tannic acid (TAA)	0.1	0.335	0.275	0.218
	0.1	0.345	0.276	0.250
	0.1	0.317	0.282	0.124
	0.1	0.162	0.154	0.052
	0.1	0.203	0.149	0.362
	0.1	0.145	0.137	0.058
	0.1	0.167	0.134	0.246
	0.1	0.157	0.152	0.033
	0.1	0.153	0.126	0.214
	1	0.296	0.269	0.100
	1	0.335	0.265	0.264
	1	0.295	0.257	0.148
	1	0.347	0.275	0.262
	1	0.272	0.276	-0.014
	1	0.336	0.282	0.191
	1	0.204	0.154	0.325
	1	0.202	0.149	0.356
	1	0.193	0.137	0.409
	1	0.209	0.134	0.560
	1	0.175	0.152	0.151
	1	0.207	0.126	0.643
	1	0.570	0.334	0.707
	1	0.516	0.584	-0.116
	1	0.338	0.279	0.211
	1	0.478	0.423	0.130
	1	0.395	0.203	0.946
	1	0.266	0.279	-0.047
theobromine (THB)	0.1	0.243	0.278	-0.126
	0.1	0.187	0.183	0.022
	0.1	0.198	0.201	-0.015
	0.1	0.218	0.201	0.085
	0.1	0.184	0.195	-0.056
	0.1	0.190	0.211	-0.100
	1	0.187	0.278	-0.327
	1	0.163	0.183	-0.109
	1	0.204	0.201	0.015
	1	0.189	0.201	-0.060
	1	0.164	0.195	-0.159
	1	0.191	0.211	-0.095
	10	0.198	0.278	-0.288
	10	0.219	0.183	0.197
	10	0.128	0.201	-0.363
	10	0.105	0.201	-0.478
	10	0.103	0.195	-0.472
	10	0.116	0.211	-0.450
	10	0.331	0.327	0.012
	10	0.309	0.332	-0.069
	10	0.295	0.378	-0.220
	10	0.326	0.295	0.105
	10	0.354	0.375	-0.056
	10	0.323	0.313	0.032
gallic acid (GAA)	0.1	0.335	0.306	0.095
	0.1	0.324	0.297	0.091

gallic acid (GAA)	0.1	0.333	0.391	-0.148
	0.1	0.343	0.299	0.147
	0.1	0.338	0.359	-0.058
	0.1	0.351	0.369	-0.049
	1	0.265	0.360	-0.264
	1	0.233	0.238	-0.021
	1	0.194	0.189	0.026
	1	0.228	0.227	0.004
	1	0.204	0.207	-0.014
	1	0.188	0.177	0.062
	10	0.224	0.218	0.028
	10	0.275	0.204	0.348
	10	0.305	0.220	0.386
	10	0.295	0.214	0.379
	10	0.233	0.218	0.069
	10	0.295	0.235	0.255
gibberellic acid (GIA)	0.1	0.308	0.348	-0.115
	0.1	0.334	0.371	-0.100
	0.1	0.367	0.359	0.022
	0.1	0.308	0.331	-0.069
	0.1	0.421	0.352	0.196
	0.1	0.281	0.347	-0.190
	1	0.314	0.396	-0.207
	1	0.390	0.469	-0.168
	1	0.403	0.443	-0.090
	1	0.387	0.391	-0.010
	1	0.372	0.365	0.019
	1	0.425	0.421	0.010
	1	0.253	0.325	-0.222
	1	0.190	0.206	-0.078
	1	0.243	0.207	0.174
	1	0.200	0.213	-0.061
	1	0.208	0.199	0.045
	1	0.236	0.176	0.341
	10	0.406	0.325	0.249
	10	0.266	0.206	0.291
	10	0.282	0.207	0.362
	10	0.316	0.213	0.484
	10	0.221	0.199	0.111
	10	0.257	0.176	0.460
